# Prior Expectations of Volatility Following Psychotherapy for Delusions: A Randomized Clinical Trial

**DOI:** 10.1001/jamanetworkopen.2025.17132

**Published:** 2025-06-24

**Authors:** Julia M. Sheffield, Ali F. Sloan, Philip R. Corlett, Baxter P. Rogers, Simon Vandekar, Jinyuan Liu, Kendall M. Beals, Lauren M. Hall, Taylor Gautier, Alexandra B. Moussa-Tooks, Lénie J. Torregrossa, Margaret Achee, Kristan Armstrong, Neil D. Woodward, Kaylee Belt, Daniel Freeman, Louise Isham, Rowan Diamond, Aaron P. Brinen, Stephan Heckers

**Affiliations:** 1 Department of Psychiatry and Behavioral Sciences, Vanderbilt University Medical Center, Nashville, Tennessee; 2 Department of Psychological Sciences, Vanderbilt University, Nashville, Tennessee; 3 Department of Psychiatry, Yale School of Medicine, New Haven, Connecticut; 4 Department of Biomedical Engineering, Vanderbilt University, Nashville, Tennessee; 5 Department of Biostatistics, Vanderbilt University, Nashville, Tennessee; 6 Department of Psychology, University of Southern Mississippi, Hattiesburg; 7 Department of Psychological and Brain Sciences, University of Louisville, Louisville, Kentucky; 8 Gautier Behavioral Health, Chattanooga, Tennessee; 9 Department of Psychological and Brain Sciences, Indiana University, Bloomington; 10 Department of Psychiatry and Behavioral Sciences, University of California, San Francisco, San Francisco; 11 Mental Health Cooperative, Nashville, Tennessee; 12 Department of Experimental Psychology, Oxford University, Oxford, United Kingdom

## Abstract

**Importance:**

Persecutory delusions are common, distressing, and difficult to treat. Testing computational neuroscience models of delusions can identify new therapeutic targets.

**Objective:**

To determine whether change in delusion severity is associated with a corresponding change in volatility priors and brain activation estimated during a belief updating task.

**Design, Setting, and Participants:**

This randomized clinical trial was conducted from April 9, 2021, to December 5, 2023, within the Vanderbilt University Medical Center Psychiatric Hospital and at a community mental health center in Nashville, Tennessee. Participants were adults (aged between 18 and 65 years) with schizophrenia spectrum or delusional disorder and an active, persistent (≥3 months) persecutory delusion with strong conviction (>50%). Participants were randomly assigned 1:1 to either cognitive behavioral therapy for psychosis (CBTp)–based intervention or befriending therapy. Intention-to-treat analysis was performed from June 1 to October 31, 2024.

**Intervention:**

The CBTp was a manualized intervention targeting persecutory delusions. The befriending therapy involved engaging in conversations and activities focused on neutral topics. Both interventions were provided in person, lasted for 8 weeks, and included standard care. Standard care consisted of medication management and ancillary services.

**Main Outcomes and Measures:**

Primary outcomes were volatility priors (ie, prior expectations of volatility) derived from a 3-option probabilistic reversal learning task; persecutory delusion severity measured by the Psychotic Symptom Rating Scales (PSYRATS delusion subscale; score range: 0-16, with the highest score indicating severe preoccupation, distress, conviction, and functioning impact); and brain activation in the striatum and prefrontal cortex measured by blood oxygenation level–dependent signal change. Associations between volatility priors, clinical improvement, and change in neural activation were examined.

**Results:**

Sixty-two participants (median [range] age, 31 [19-63] years; 38 males [61%]) were randomly assigned to the CBTp (n = 32) or befriending therapy (n = 30) arms. A subgroup of 35 participants (57%) completed functional magnetic resonance imaging. Volatility priors decreased following treatment (*F*_1,112_ = 7.7 [*P* = .006]; Cohen *d* = 0.52 [95% CI, 0.15-0.90]), as did delusion severity (*F*_1,112_ = 59.7 [*P* < .001]; Cohen *d* = 1.50 [95% CI, 1.00-1.90]), across both groups. The decrease in volatility priors was not associated with clinical improvement in PSYRATS scores (*F*_1,102.8_ = 1.8 [*P* = .18]; Cohen *d* = 0.26 [95% CI, –0.12 to 0.65]). Activation in the caudate and prefrontal cortex significantly decreased following treatment. Decreased caudate activation was associated with decreased volatility priors (*F*_1,58.3_ = 16.6 [*P* < .001]; Cohen *d* = 1.07 [95% CI, 0.51-1.61]) but not with PSYRATS total scores. Associations remained significant after controlling for antipsychotic medication (*F*_1,53_ = 13.77; *P* < .001).

**Conclusions and Relevance:**

This randomized clinical trial found that elevated volatility priors and associated activation in the caudate nucleus were amenable to change. Volatility priors could be a potential target for intervention in psychosis.

**Trial Registration:**

ClinicalTrials.gov Identifier: NCT04748679

## Key Points

**Question:**

Do prior expectations of environmental volatility (ie, volatility priors) and associated neurobiological correlates change with treatment of delusions?

**Findings:**

In this randomized clinical trial of 62 participants with schizophrenia spectrum disorder and severe and persistent persecutory delusion, volatility priors and associated activation in the caudate nucleus decreased following psychotherapy.

**Meaning:**

The findings suggest that volatility priors are amenable to change with treatment and may be a novel target for intervention in psychosis.

## Teaser

his randomized clinical trial examines the association between volatility expectation and psychotic symptom severity among adults with schizophrenia spectrum or delusional disorder receiving treatment for persecutory delusion.

## Introduction

Delusional beliefs are hallmark symptoms of psychotic disorders that contribute to marked distress and disability.^1,2^ Persecutory delusions—the belief that others intend one harm—are the most common form of delusion, present in more than 70% of individuals with psychosis.^3^ Despite the demonstrated effectiveness of medications and psychotherapy in managing delusions, many individuals continue to experience them.^4,5^ Advancing treatments will be aided by a more precise identification of disrupted processes that contribute to delusion severity.^6,7,8,9^

Interventionist approaches to psychiatry urge the use of randomized clinical trials (RCTs) as a tool for establishing causal inferences about hypothesized mechanisms.^10^ As fields such as computational psychiatry, neuroscience, and psychology identify processes associated with psychiatric symptoms, RCTs are needed to elevate the status of these processes as a causal mechanism deserving of targeted treatment.^11^

One such candidate mechanism for persecutory delusions is elevated volatility priors (ie, prior expectations of volatility). Mounting evidence suggests that individuals with high paranoia and persecutory delusions expect more volatility in their environment,^12^ reflecting a belief that the world is frequently changing. Volatility priors^13,14,15,16^ are associated with persecutory delusion severity in schizophrenia,^17^ correlate with intensity of childhood maltreatment,^18^ and track with paranoia in the general population.^16^ Phenomenologically, volatility is core to the lived experiences of individuals with persecutory delusions. The paranoid style has been described as living “constantly ... at a turning point,”^19^ and historical accounts of delusional thinking describe a global impression that “something unknown is going on”^20^^p(88)^ and therefore things cannot be “taken for granted.”^21^^p(300)^ While these phenomena have been described for decades, computational psychiatry provides a method for measuring these expectations of environmental change.

Volatility priors are an aspect of the broader predictive coding model of psychosis,^22^ which views belief updating as fundamental to delusion development and maintenance.^22,23^ Belief updating is the process of integrating prior expectations with incoming information to better understand and anticipate one’s environment.^24^ Whether and how beliefs are updated is impacted by the volatility of the environment or how frequently the environment changes.^13,25^ Accurately estimating volatility is crucial for mental health. Overestimation of volatility renders likely outcomes as overly salient, promoting the formation of beliefs based on faulty inference (as is observed with delusions).^24,26^

Furthermore, belief updating is instantiated in known brain mechanisms that involve the striatum and prefrontal cortex (PFC).^27^ The PFC is critical for learning and decision-making,^28^ and elevated volatility priors are associated with increased dorsolateral PFC activation in schizophrenia^29^ and individuals at clinical high risk.^30^ The PFC is connected to the striatum and is implicated in the pathophysiological processes of psychosis.^31^ The associative striatum, including the caudate nucleus, is densely innervated by midbrain dopamine neurons that signal salience of new information.^28,32,33^ Increased activation in the associative striatum may reflect inappropriate attribution of salience, promoting an overestimation of volatility.

In line with interventionist models of psychiatry, we conducted an RCT of psychotherapy to determine whether change in delusion severity is associated with a corresponding change in volatility priors and brain activation estimated during a belief updating task. We hypothesized that (1) effective treatment of delusions would decrease volatility priors and activation in the striatum and PFC and (2) changes in volatility priors and brain activation would correlate to changes in severity of delusions.

## Methods

### Study Design

A parallel, assessor-blind RCT was conducted at Vanderbilt University Medical Center from April 9, 2021, to December 5, 2023. Recruitment of participants occurred within the Vanderbilt Psychiatric Hospital and at a community mental health center in Nashville, Tennessee. The trial received ethical approval from the Vanderbilt Institutional Review Board. All participants provided written informed consent. The full trial protocol is available in Supplement 1. We followed the Consolidated Standards of Reporting Trials (CONSORT) reporting guideline.

### Participants

Eligibility criteria included patient age 18 to 65 years; diagnosis of schizophrenia, schizoaffective disorder, schizophreniform disorder, or delusional disorder; persecutory delusion present for at least 3 months with more than 50% conviction; Penn State Worry Questionnaire (PSWQ)^34^ score greater than 43 (score ranges: 44-62 [indicating moderate worry] and ≥63 [indicating high worry]); and sufficient English-language skills for participation. Exclusion criteria included premorbid IQ lower than 70 as measured by the Wechsler Test of Adult Reading^35^ (score range: 50-131, with the highest score indicating superior estimated premorbid IQ); traumatic brain injury; lifetime loss of consciousness for more than 30 minutes; epilepsy or other neurological disorder; and ongoing use of substances (other than nicotine and marijuana). Diagnosis was determined using the Structured Clinical Interview for the *Diagnostic and Statistical Manual of Mental Disorders* (Fifth Edition) or by the participant’s outpatient psychiatrist and medical record review. Race and ethnicity were self-reported by participants and analyzed to provide an overview of the sample for generalizability of findings. A list of options for race, created by the study team based on US Census Bureau categories and National Institutes of Health reporting guidelines, was provided to participants to choose from. Race and ethnicity categories included in the analysis were American Indian or Alaska Native, Black or African American, White, multiracial, and other (which was not further defined by the study team).

### Randomization and Masking

**Figure 1.**
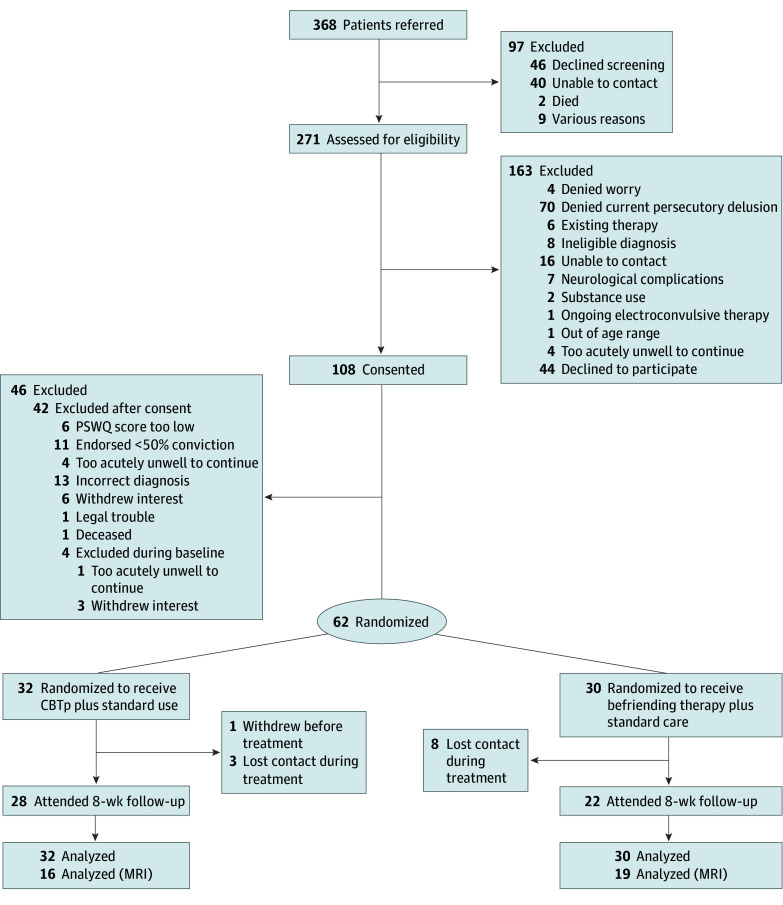
Trial Flow Diagram CBTp indicates cognitive behavioral therapy for psychosis; MRI, magnetic resonance imaging; PSWQ, Penn State Worry Questionnaire.

Participants were randomly assigned 1:1 to either a manualized cognitive behavioral therapy for psychosis (CBTp)–based intervention for persecutory delusions or befriending therapy for 8 weeks. Both arms also allowed for standard care consisting of medication management and ancillary services (Figure 1). Randomization was based on an algorithm developed by the study biostatistician (S.V.), with stratification by worry severity (using PSWQ scores) and randomly varying block sizes (4-6). Study assessors (K.M.B., L.M.H., and A.F.S.) were masked to allocation, and breaks in masking were recorded. If unmasking occurred, reallocation to another rater was completed when feasible.

### Interventions

Details on the interventions are included in the eMethods in Supplement 2. Briefly, the manualized CBTp for persecutory delusions intervention was developed by Freeman and colleagues.^36^ The befriending therapy involved engaging in conversations and activities focused on neutral topics.^37^ Both treatments were conducted in person for approximately 50 minutes by 1 of 5 trained doctoral-level psychologists (A.P.B., J.M.S, A.B.M-T, L.J.T, and M.A.) or by 1 trained, licensed clinical social worker (T.G.). Training for both treatments was conducted by the Oxford Cognitive Approaches to Psychosis research group (D.F., L.I., and R.D.) and supervised by a psychologist (A.P.B). All sessions were recorded for fidelity, which was assessed by independent raters.

### Measurements

Clinical and cognitive assessments, including functional magnetic resonance imaging (fMRI), were performed at baseline and end of treatment (at week 8). Blinded assessors conducted assessments in a research suite at Vanderbilt Psychiatric Hospital. All participants completed the same belief updating task. Thirty-five MRI-eligible participants completed the task during fMRI (eTable 2 in Supplement 2), while all other participants completed it in the research suite. During fMRI, the blood oxygenation level–dependent (BOLD) signal, which approximates neural activity during performance of a cognitive task, was collected. This information allowed for measurement of changes in regional activation before and after treatment.

### Task and Computational Modeling

At their baseline (pretreatment) and posttreatment assessments, participants completed a 3-option probabilistic reversal learning (3-PRL) task^14,15,16,17^ (eFigure 1 in Supplement 2), which assessed belief updating in the context of a volatile environment. During the 3-PRL task, participants were presented with 3 decks of cards on a computer screen and instructed to find the best deck (rewarded the most frequently). Reward or loss feedback was a gain of 100 points or a loss of 50 points. Volatility was embedded in the task in 2 ways: (1) the best deck changed after the participant selected it 9 out of 10 times in a row, and (2) the underlying reward contingencies for each deck (eg, if the decks rewarded participants 90%, 50%, or 10% of the time) changed halfway through the task. These changes occurred without knowledge of the participant, increasing uncertainty and volatility.

Volatility priors were computationally modeled using a hierarchical Gaussian filter, as previously reported.^14,15,16,38,39^ The primary parameter of interest was μ^0^_3_, which captured prior beliefs about environmental volatility, reflecting how much the participant expected the task environment to change. Higher μ^0^_3_ reflected greater expectations of volatility. Details and parameter recovery are provided in the eMethods and eFigure 2, respectively, in Supplement 2.

### Neuroimaging Analysis

A subset of participants performed the 3-PRL task while in an MRI scanner, providing an approximation of brain activity during belief updating (eMethods in Supplement 2). Briefly, task trials were divided into a decision period (time between when the cards were seen and a deck was selected) and a feedback period (time between when reward or loss feedback was received and the next fixation cross was presented). We expected activation during the decision period to be most relevant for associations with volatility priors, as this time is when prior expectations should be influencing deck choice. To test for activation in the a priori regions of interest (striatum and PFC), we initially masked (ie, limited) data analysis to parts of the striatum and PFC that were substantially activated by the task (eFigure 3 in Supplement 2). We then explored changes in activation before vs after treatment across the whole brain. ^40,41^

### Outcomes

Primary outcomes included volatility priors (μ^0^_3_) as derived from the 3-PRL task; persecutory delusion severity as measured by the Psychotic Symptom Rating Scales (PSYRATS); and brain activation in the striatum and PFC as indicated by the BOLD signal. The PSYRATS^42^ delusions subscale assesses the severity of a specific delusion (score range: 0-16, with the highest score indicating severe preoccupation, distress, conviction, and functioning impact). This delusion was rated by the participant at more than 50% conviction at the start of the study and then rerated at subsequent assessments. The primary belief updating metric of interest was μ^0^_3_. Primary fMRI outcomes included BOLD signal change in the striatum and PFC during the decision phase of the task.

Secondary outcomes included sensitivity to volatility (κ) and meta-volatility learning rate (ω_3_) (eMethods in Supplement 2). The Positive and Negative Syndrome Scale (PANSS) positive symptom subscale^43^ (score range: 7-49, with the highest score indicating extremely severe psychotic symptoms) was used to capture a broader picture of psychosis symptom severity.

### Statistical Analysis

Volatility parameters were examined for normality and log transformed to address skew in the data using the optLog package in R (R Project for Statistical Computing). Three linear mixed models, with participant as a random effect, were used to test our hypotheses: (1) outcome (cognitive or clinical) as the dependent variable, with time, treatment, baseline outcome, MRI (yes or no), and treatment-by-time interactions as fixed effects; (2) clinical outcome as the dependent variables, with treatment and volatility parameters as fixed effects; and (3) clinical or cognitive outcome as the dependent variable, with treatment and BOLD activation as fixed effects.

Despite a priori hypotheses, 2-sided significance was reported for rigor. Hypotheses were tested using type 2 sum of squares so that main effects were tested without interaction terms in the model. Power analysis was conducted using the simr package in R to simulate data from a linear mixed-effects model assuming 2-sided significance (α = .03), a sample size of 60, and 15% attrition of data throughout the study. Under these assumptions, we had 84% power to detect an effect size of 0.6. Effect sizes were calculated using linear mixed models (*F* statistic) and converted to Cohen *d* effect size using the effectsize package in R without a paired assumption. Pearson *r* was used for correlations, and χ^2^ tests assessed differences in categorical variables. Primary outcomes were uncorrected for multiple comparisons; however, analyses of PANSS positive symptoms were considered significant at *P* < .025 (Bonferroni correction). Intention-to-treat analysis was performed from June 1 to October 31, 2024.

## Results

From April 9, 2021, to December 5, 2023, 271 individuals were assessed for eligibility, of whom 108 consented to participate and 62 were randomly assigned to receive CBTp (n = 32) or befriending therapy (n = 30) and included in the final intent-to-treat analysis (Table; Figure 1). These participants included 24 females (39%) and 38 males (61%), with a median (range) age of 31 (19-63) years. Three participants (5%) self-identified as American Indian or Alaska Native, 24 (39%) as Black or African American, 30 (48%) as White, 2 (3%) as multiracial, and 3 (5%) as other race and ethnicity. Posttreatment data were missing for 12 individuals who withdrew prior to treatment start (n = 1) or lost contact with the study team during treatment (n = 11). Rates of medication change during treatment were similar across the CBTp and befriending therapy groups (23% and 27%; χ^2^ = 0.66; *P* = .77).

**Table.** Patient Demographics

Characteristic	Participants, No. (%)	
	CBTp (n = 32)	Befriending therapy (n = 30)
Age, mean (SD), y	36.0 (13.8)	30.9 (8.8)
Sex		
Female	13 (41)	11 (37)
Male	19 (59)	19 (63)
Race and ethnicity^a^		
American Indian or Alaska Native	1 (3)	2 (7)
Black or African American	13 (41)	11 (37)
White	15 (47)	15 (50)
Multiracial	1 (3)	1 (3)
Other^b^	2 (6)	1 (3)
Years of personal education, mean (SD)	13.3 (2.4)	14.0 (2.6)
Years of parental education, mean (SD)	14.0 (2.7)	14.8 (2.9)
Premorbid IQ, mean (SD)^c^	98.1 (13.8)	99.3 (16.7)
Cognitive ability score, mean (SD)^d^	−1.5 (0.9)	−1.3 (1.3)
Risperidone equivalence, mean (SD)	5.7 (4.6)	6.7 (5.3)

Abbreviation: CBTp, cognitive behavioral therapy for psychosis.

^a^ Race and ethnicity were self-reported by participants and analyzed to provide an overview of the sample for generalizability of findings.

^b^ Participants were given the option to select Other as their race, but this was not further defined by the study team. A list of options for race, created by the study team based on US Census Bureau categories and National Institutes of Health reporting guidelines, was provided to participants to choose from. Only categories included in the analysis appear in the table.

^c^ Premorbid IQ was assessed with the Wechsler Test of Adult Reading tool.

^d^ Cognitive ability was assessed using the Screen for Cognitive Impairment in Psychiatry tool. *Z* scores are presented compared with a sample of healthy adults.

**Figure 2.**
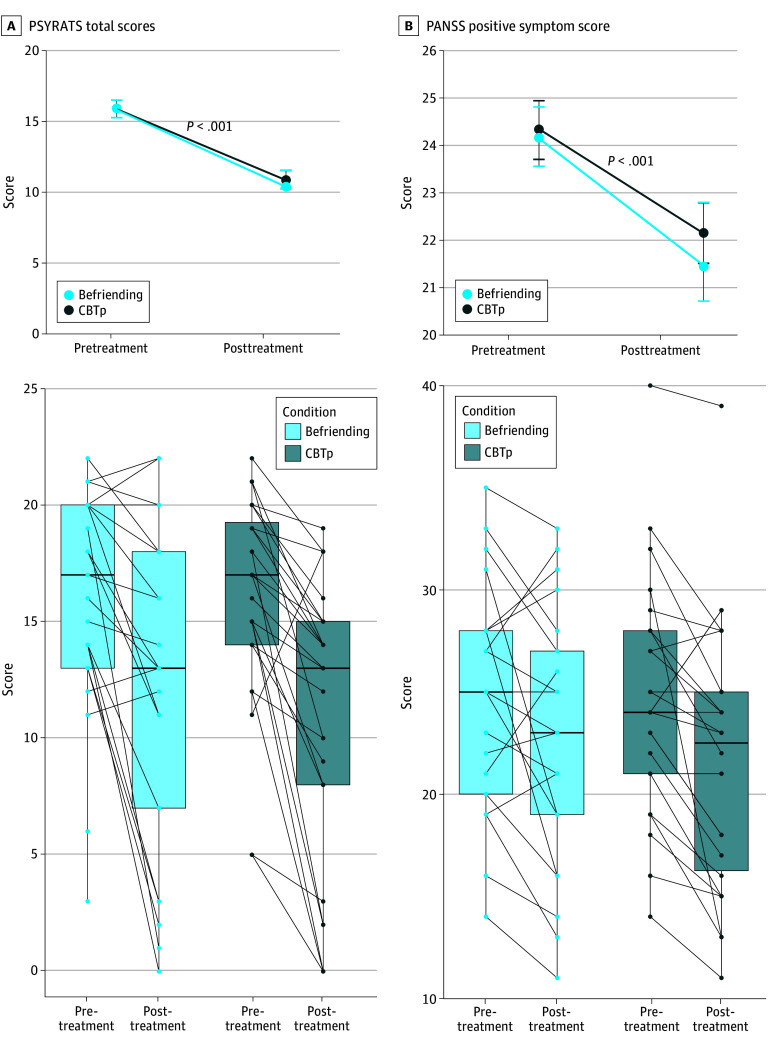
Change in Clinical Outcomes With Psychotherapy CBTp indicates cognitive behavioral therapy for psychosis; PANSS, Positive and Negative Syndrome Scale, Positive Subscale; PSYRATS, Psychotic Symptom Rating Scales.

Clinical outcomes are presented in eTable 1 in Supplement 2. PSYRATS total scores decreased in both conditions as evidenced by a main effect of time (*F*_1,112_ = 59.7 [*P* < .001]; Cohen *d* = 1.50 [95% CI, 1.00-1.90]) but a nonsignificant treatment-by-time interaction (*F*_1,112_ = 0.06; *P* = .81) (Figure 2A). Similarly, PANSS positive scores decreased (*F*_1,113_ = 14.7 [*P* < .001]; Cohen *d* = 0.72 [95% CI, 0.34-1.10]), but the treatment-by-time interaction was nonsignificant (*F*_1,113_ = 0.17; *P* = .68) (Figure 2B).

**Figure 3.**
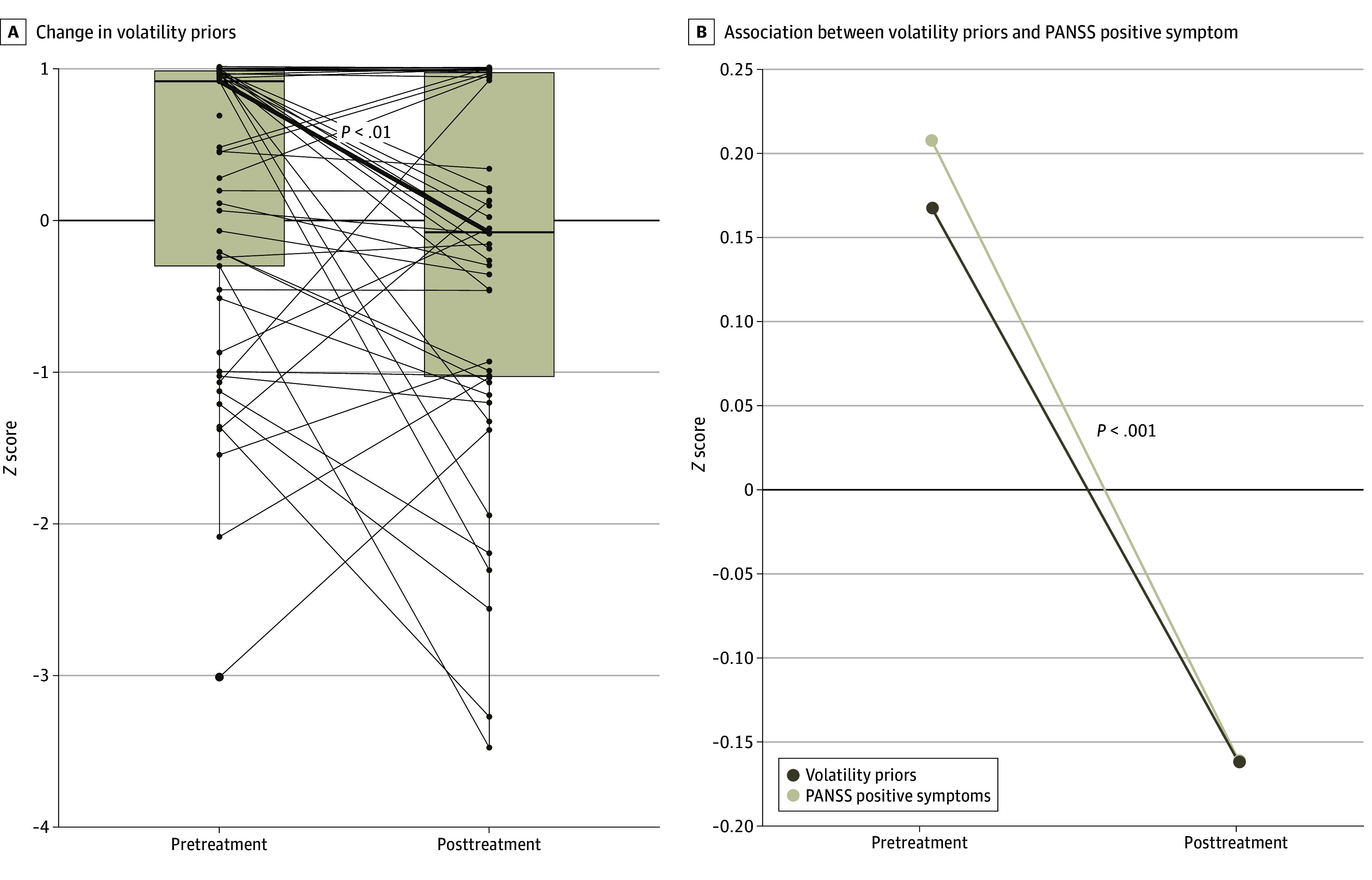
Change in Volatility Priors With Psychotherapy and Association With Positive and Negative Syndrome Scale (PANSS) Positive Symptoms

Across both treatment groups, volatility priors significantly decreased (*F*_1,112_ = 7.7 [*P* = .006]; Cohen *d* = 0.52 [95% CI, 0.15-0.90]) (Figure 3A). There was no significant main effect of treatment (*F*_1,112_ = 0.23 [*P* = .63]; Cohen *d* = 0.09 [95% CI, −0.28 to 0.46]) or treatment-by-time interaction (*F*_1,112_ = 0.06; *P* = .80).

### Associations Between Clinical Outcomes and Volatility Priors

Across all participants, volatility priors were not associated with clinical improvement in PSYRATS scores (*F*_1,102.8_ = 1.8 [*P* = .18]; Cohen *d* = 0.26 [95% CI, –0.12 to 0.65]). Volatility priors, however, were associated with clinical improvement in PANSS positive symptoms scores (*F*_1, 110.7_ = 11.7 [*P* < .001]; Cohen *d* = 0.65 [95% CI, 0.27-1.03]) (Figure 3B). Associations with specific PANSS items are reported in the eResults in Supplement 2.

### fMRI Results 

**Figure 4.**
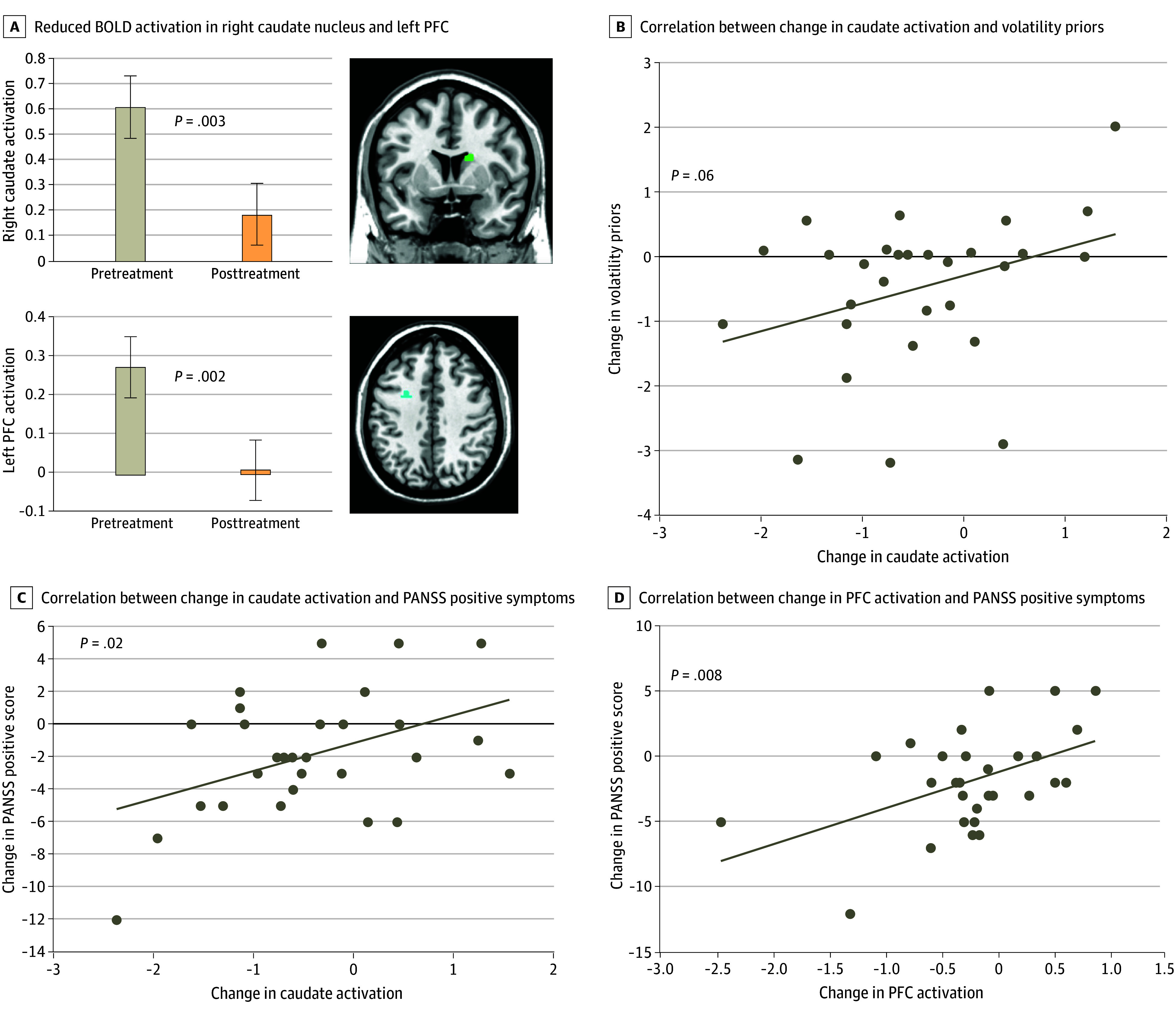
Change in Functional Magnetic Resonance Imaging Activation and Association With Volatility Priors and Positive and Negative Syndrome Scale (PANSS) Positive Symptoms Error bars represent SEs of the mean. BLOD indicates blood oxygenation level–dependent; PFC, prefrontal cortex.

Thirty-five participants (57%) completed fMRI. Two clusters exhibited significantly decreased activation after treatment: a region in the right caudate (κ = 17; *P* = .003) and another in the left PFC (κ = 22; *P* = .002) (Figure 4A). Activation in these regions was significantly decreased from before to after treatment (caudate: *F*_1,34.2_ = 5.6 [*P* = .02]; Cohen *d* = 0.81 [95% CI, 0.11-1.50]; left PFC: *F*_1,64_ = 5.7 [*P* = .02]; Cohen *d* = 0.64 [95% CI, 0.09-1.10]).

Decreased caudate activation was associated with change in volatility priors across all participants (*F*_1,58.3_ = 16.6 [*P* < .001]; Cohen *d* = 1.07 [95% CI, 0.51-1.61]), and changes in scores were positively correlated (*r* = 0.34; *P* = .06) (Figure 4B). By contrast, the region in the left PFC was not associated with volatility priors (*F*_1,54_ = 1.6 [*P* = .21]; Cohen *d* = 0.34 [95% CI, −0.19 to 0.88]).

Decreased caudate activation was not associated with PSYRATS total scores (*F*_1,57.5_ = 0.97 [*P* = .33]; Cohen *d* = 0.26 [95% CI, −0.26 to 0.78]) but was associated with PANSS positive symptoms (*F*_1,34.1_ = 7.2 [*P* = .01]; Cohen *d* = 0.92 [95% CI, 0.21-1.62]). Change in PANSS positive symptoms correlated with change in caudate activity (*r* = 0.43; *P* = .02) (Figure 4C).

Decreased left PFC activation was not associated with PSYRATS scores (*F*_1,55.3_ = 3.36 [*P* = .07]; Cohen *d* = 0.49 [95% CI, −0.04 to 1.03]) but was associated with PANSS positive symptoms (*F*_1,35_ = 15.4 [*P* < .001]; Cohen *d* = 1.33 [95% CI, −0.59 to 2.05]). Change in left PFC activation was correlated with change in PANSS positive symptoms (*r* = 0.48; *P* = .008) (Figure 4D). Analyses conducted for sensitivity and specificity are described in detail in the eResults in Supplement 2.

### Healthy Comparison Participants

A group of healthy comparison participants (n = 27) completed the 3-PRL task twice in the MRI scanner, 8 weeks apart, as part of another study. These participants demonstrated no significant change in volatility priors or activation in the caudate or left PFC over 8 weeks (eFigure 4 in Supplement 2).

### Other Psychopathological Processes and Medication

Volatility priors were not associated with change in depression (*F*_1,108.8_ = 1.0; *P* = .31), negative symptoms (*F*_1,108.2_ = 1.2; *P* = .27), or general symptoms (*F*_1,110.7_ = 1.7; *P* = .68). All primary analyses were conducted, controlling for antipsychotic dose at the time of the assessment (risperidone equivalence).^44^ Results continued to be significant when controlling for medication—for example, the association between volatility priors and caudate activation (*F*_1,53_ = 13.77; *P* < .001).

### Thresholding and Whole-Brain Analysis

Within the regions of interest, we examined changes in activation at a lower, exploratory threshold (κ>15; *P* < .01). Results suggest reduced activation in the left caudate and right dorsolateral PFC after treatment (eResults in Supplement 2). In addition, we explored the whole brain for regions showing significantly reduced activation with treatment (eTable 3 in Supplement 2). Of these regions, reduced activation in the cerebellum and hippocampus was associated with decreased volatility priors (cerebellum: *F*_1,59_ = 8.1 [*P* = .006]; hippocampus: *F*_1,50.3_ = 8.5 [*P* = .005]) and decreased positive symptoms (cerebellum: *F*_1,38.5_ = 8.3 [*P* = .006]; hippocampus: *F*_1,33.1_ = 6.8 [*P* = .01])

## Discussion

This RCT of psychotherapy examined changes in volatility beliefs, a putative cognitive mechanism of delusions, in schizophrenia spectrum disorders.^10^ Prior expectations about environmental volatility decreased with psychological treatment, and these reductions were associated with clinical improvement in PANSS positive symptom severity and decreased activation in the caudate nucleus. Sensitivity analyses revealed that these relationships (1) did not extend to other clinical phenomena, such as negative symptoms, general psychopathological process, and depression; (2) were robust to the inclusion of antipsychotic medication dose; and (3) were not observed in healthy comparison participants assessed longitudinally. These findings add to the growing literature that leverages computational psychiatry to demonstrate how specific alterations in belief updating contribute to psychotic experiences.^45,46,47,48^

Primary analysis of clinical data focused on the PSYRATS, a tool that assesses the severity of a specific delusion elicited in collaboration with the participant. Psychotherapy supported a large improvement in delusion severity after only 8 weeks, yet associations with volatility parameters and task-evoked brain activation were not statistically significant. Instead, associations were observed with overall positive symptom severity as measured by PANSS. Exploratory analyses revealed volatility priors related to PANSS delusion items but not hallucinations, indicating some specificity to delusions. However, previous work has suggested a relatively specific association between volatility priors and persecutory delusions or paranoia,^17,49^ including the PANSS item suspiciousness or persecution, which was not observed in the present study. Those findings were reported in nonclinical samples,^15^ clinical high-risk populations,^49^ and stable outpatients with very low delusion severity.^14^ The current study, by contrast, included highly symptomatic patients with strongly held persecutory delusions, which likely involved more complex themes than lower-level paranoia. In this RCT, volatility priors may therefore be tracking a general loosening of the delusional system and improvement in psychotic experiences, which includes a strongly held persecutory belief. In addition, multiple cognitive and psychological factors contribute to delusions.^50^ Our results suggest that volatility priors may be an additional mechanism, situated at a level of analysis closer to neurobiology that can be targeted in treatment,^51^ bridging neuroscience and clinical application.^52^

To our knowledge, this RCT was the first in over a decade^53^ to examine task-based fMRI before and after psychotherapy for schizophrenia. Overall, substantial changes in brain response were found following psychological treatment. Given that participants in both treatment groups showed improvement, we cannot definitively say that activation changes were caused by the treatment; however, healthy comparison participants did not demonstrate similar changes, suggesting the changes were not due to practice effects or familiarity with the task environment.

In line with our hypotheses, activation in the caudate nucleus decreased during belief updating and was associated with reduced volatility priors and positive symptom severity. The caudate is part of the associative striatum^31,54^ and is the locus of an elevated level of presynaptic dopamine in schizophrenia.^55^ During reversal learning, striatal activity maps closely to dopamine release, signaling salience.^56^ The observed reduction in caudate activation may therefore reflect less precocious salience signaling after treatment,^57,58^ thereby improving positive symptom severity. A similar reduction in activation was observed within the PFC, which was also related to positive symptom severity. Within the associative pathway, the caudate receives afferent connections from the PFC, and strengthening of caudate-PFC functional connectivity following antipsychotic treatment correlates with improvement in positive symptom severity in schizophrenia.^59^ Signals of change that correlated with volatility and positive symptoms were also observed in the hippocampus and cerebellum, both of which are critical nodes in psychosis pathophysiological process.^60,61,62^

### Limitations

Limitations of this trial include similar treatment response in the CBTp and befriending therapy arms, limiting causal inferences about change in volatility. Active comparison therapies often perform well in trials,^63^ and befriending may have served as an implicit social exposure, improving paranoia. This RCT, while adequately powered for moderate effects, had relatively low sample sizes, particularly in the MRI analysis.

## Conclusions

This RCT presented evidence to support volatility priors as a contributor in psychotic symptom severity, adding to the expanding literature on belief updating and predictive coding models of delusions. Volatility priors could be a potential target for intervention in psychosis. Future studies are warranted to examine these associations in a larger trial, the factors that contribute to elevated volatility priors (eg, unpredictability during childhood^18^ and racial discrimination^64^), and the development of treatments targeting volatility expectations.

## Notes

Supplement 1.
Trial Protocol

Supplement 2.
eMethods

eResults
**eTable 1.** Outcomes **eTable 2.** MRI Cohort **eTable 3.** Significant Clusters in Whole Brain Analysis **eFigure 1.** A) Task, B) Decision and Feedback-Periods Defined for fMRI Analysis, C) HGF Model **eFigure 2.** Parameter Recovery **eFigure 3.** Masks Used **eFigure 4.** Healthy Comparison Group

Supplement 3.
Data Sharing Statement
