# Supplementary material for: Prior Expectations of Volatility Following Psychotherapy for Delusions: A Randomized Clinical Trial
Source: JAMA Netw Open. 2025 Jun 24;8(6):e2517132. doi: 10.1001/jamanetworkopen.2025.17132 (PMC12188364; doi:10.1001/jamanetworkopen.2025.17132)
Supplement: Supplement 1. — Trial Protocol [file jamanetwopen-e2517132-s001.pdf]

Cognitive Processes Underlying Psychotherapy for Persecutory Delusions

Julia Sheffield, PhD
Assistant Professor
Department of Psychiatry and Behavioral Sciences
Vanderbilt University Medical Center
1601 23rd Ave. S Suite 3060
Nashville, TN 37212

Amendment History

Original Submission: 9/20/2020

Amendment 1 (2/12/21): addition of 2 new self-reports and 4 new cognitive tasks and removal of 2 cognitive tasks. Change in compensation for participants. Change of “befriending therapy” to “connection therapy” in participant materials. Addition of IRB#191321 as a recruitment source.

Amendment 2 (3/17/21): This amendment aims to add one clinical interview (Time Use Survey) and two self-reports (Working Alliance Inventory, Credibility/Expectancy Questionnaire). We also add language to the application, ICD and protocol to allow for video-taping of clinical interviews. This video-taping will allow for the assessment of inter-rater reliability as additional assessors are trained and included in the study. This is particularly important if our primary rater becomes unblinded following randomization, in order to reduce bias. Finally, we have edited our screening forms (Phone Screening Script and Screening Form). These forms have been edited to inform participants that the information they provide during the screening that determines their eligibility for the study will be stored in a password protected spreadsheet. In addition, we have made more minor edits to the screening form following review of the language used in the original form.

Amendment 3 (8/23/21): We have changed the phone number on the recruitment/ICD/Screening forms due to researchers working with a new phone. We have added one document to the recruitment section of document uploads (Advertisement). This document is a flyer specifically for prospective participants. We have added a resource for Outpatient Therapy Referrals, that participants can use to identify therapists in the community if they want to continue with therapy. We have also included a self-report measure of emotion regulation. We have added in language the application to include text as a form of communication (in addition to the existing call/email). We have added information to the recruitment section about recruiting individuals from the community. Lastly we have changed the payment schedule of paying participants $20 for a diagnostic interview to $15 per hour.

Amendment 4 (2/15/22): We had not updated our protocol in line with recent IRB amendments and so an updated protocol is attached. Furthermore in line with similar studies, we have added language into our application that includes the collection of data from other psychiatry studies that assess similar attributes in order to decrease the burden for participants. We have also added Negative Valence Systems in Schizophrenia (IRB: 211116) to our recruitment section in order to open up the ability to recruit participants from their participant pool. We have added a video to aid with recruitment. This video uses verbatim language from approved IRB documents. Lastly, we have increased the accrual goal to 150 participants.

Amendment 5 (7/6/22): Change of study coordinator

Amendment 6 (8/24/22): We have updated our exclusion criteria to reflect the fact that individuals who have conditions that preclude MR scanning will not complete the imaging portion of the study, but are otherwise able to participate in the study. We have amended the IRB, protocol, and consent document to reflect that ineligibility for MR scanning is not an exclusion from the study in its entirety. We have amended the IRB to be sure the IRB and participants are informed that the 3-PRL task completed during the week 4 and week 24 visits is run through a HIPPA-compliant server hosted by Yale University, which was not explicitly stated previously. We have added the Body, Mind and Brain Lab at Vanderbilt as a recruitment source. We have added the Collaborative Design for Recovery and Health as a recruitment source and added a document outlining the language that will be sent out to a 3000 member group. Finally, we are adding two self-report measures to assess perceived discrimination.

Amendment 7 (1/30/23): We have updated our recruitment efforts to include a new recruitment flyer, Facebook as a recruitment source, and a Facebook post to be used. Additionally, we have updated the WIT

and Connection therapy fliers to reflect a change in study personnel. We have also updated the study protocol to support the diagnostic interview being optional in the case that an individual participant's diagnosis can be determined by review of their medical record. Lastly, we have updated the application to reflect the fact that individual participants can request information from the research study to be provided to their treating physician.

Table of Contents:

Study Schema
1.0 Background
2.0 Rationale and Specific Aims
3.0 Inclusion/Exclusion Criteria
4.0 Enrollment/Randomization
5.0 Study Procedures
6.0 Reporting of Adverse Events or Unanticipated Problems involving Risk to Participants or Others
7.0 Study Withdrawal/Discontinuation
8.0 Statistical Considerations
9.0 Privacy/Confidentiality Issues
10.0 Follow-up and Record Retention

1.0 BACKGROUND

Persecutory delusions, a fixed belief of deliberate threat, are present in more than 80% of persons during a first episode of psychosis. Persecutory delusions increase risk of suicide, violence, and hospitalization, underscoring the need for effective treatments. Unfortunately, the cognitive and neurobiological mechanisms underlying persecutory delusions are poorly understood and under-investigated, stalling treatment advancement. To address this critical knowledge gap, a randomized-controlled trial (RCT) will be conducted to test whether psychotherapy influences a proposed cognitive mechanism underlying persecutory delusions: abnormal belief updating. Belief updating is the process of integrating new information into existing beliefs systems, and inappropriate belief updating is suggested as a core contributor to delusion severity. Recently, a large-scale clinical trial of 146 psychotic disorder patients with persecutory delusions demonstrated that those who received a brief (6-session), manualized intervention (Worry Intervention Training (WIT)) showed a significantly greater reduction in delusion severity than patients receiving standard care (Freeman et al., Lancet Psychiatry, 2015). Despite its efficacy, the cognitive and neurobiological basis of this intervention has not been tested. This proposal will test the hypothesis that abnormal belief updating is a mechanism underlying persecutory delusion severity in psychotic disorder patients that is normalized by WIT psychotherapy. If so, we expect that individuals in the WIT condition will demonstrate significantly greater changes in belief updating over time than individuals receiving Befriending therapy - a supportive psychotherapy that controls for the general factors of therapy, such as weekly engagement and a working alliance. The project will enroll patients with persecutory delusions (N=60) into an RCT of WIT (N=30) or Befriending (N=30) to assess belief updating behaviorally and with fMRI pre/post treatment. The goals of the project are to determine the role of belief updating in persecutory delusion severity and identify neural targets of belief updating changes pre/post treatment, in order to guide future treatment development.

2.0 RATIONALE AND SPECIFIC AIMS

Persecutory delusions, a fixed belief of deliberate threat, cause significant emotional distress, lead to social isolation, and heighten risk of violence to self and others (Coid et al., 2013). The majority of persons in an early stage of psychosis present with persecutory delusions, contributing to increased incarceration, hospitalization, and poorer quality of life. Persecutory delusions are also present in multiple psychiatric disorders (e.g. schizophrenia-spectrum, affective disorders), yet current anti-psychotic and psychotherapeutic treatments yield small to moderate effects on delusion severity. Poor understanding of the cognitive and neural mechanisms underlying persecutory delusions is stalling treatment advancement.

Theoretical models of persecutory delusions posit abnormal Bayesian belief updating as a core cognitive mechanism underlying their formation and maintenance (Fletcher and Frith, 2009). When people form beliefs, they weigh their prior expectations against incoming sensory information. A mismatch between the two leads to a prediction error, which drives updating of beliefs about the environment. Delusions are hypothesized to be caused by an imbalance between sensory experiences and prior expectations, resulting in abnormally strong prediction errors (Sterzer et al., 2018). This elevates the experience of volatility – the tendency towards inferring sudden changes in the environment based on sensory data. Volatility scales the belief update, meaning that highly volatile environments lead to more rapid belief updates. Less ideal (i.e. overly rapid) Bayesian belief updating is correlated with greater paranoid ideation in healthy individuals(Nour et al., 2018). Yet, it is unknown whether changes in belief updating contribute to changes in the severity of persecutory delusions in psychotic disorder patients. Critically, recent advances in computational psychiatry have made it possible to measure belief updating parameters (e.g. volatility) using relatively simple behavioral tasks, paving the way for clinical application in psychotic disorders. Individuals with schizophrenia, as well as those in the general population reporting heightened paranoia, exhibit elevated volatility relative to healthy or low-paranoia

individuals (Deserno et al., 2020; Reed, et al., 2020). Testing whether changes in belief updating track with changes in persecutory delusion severity in a randomized-controlled trial (RCT) will validate belief updating as a novel treatment target.

Belief updating activates a known network of regions that form its neurobiological basis. In healthy adults, this network includes the ventral striatum, insula, and dlPFC (Deserno et al., 2020; Nour et al., 2018; Powers et al., 2017). All of these regions have shown functional abnormalities in psychosis and their activation during belief updating covaries with volatility estimates (Deserno et al., 2020). In healthy subjects, striatal activation during encoding of belief updates is strongly associated with striatal dopamine release capacity and availability (Nour et al., 2018). The insula is the hub of the salience network that activates in response to prediction errors (Preuschoff et al., 2008) and is also associated with dopaminergic measures(McCutcheon et al., 2019). Relationships with dopamine strengthen this network's relevance to psychosis, as dopaminergic dysfunction is hypothesized as a core feature of psychotic disorders and experiences. In schizophrenia, elevated volatility is associated with increased dlPFC activation in medicated and unmedicated patients relative to healthy controls (Deserno et al., 2020). Investigating how activation of these regions changes throughout an RCT will reveal a neurobiological basis of treatment targets.

Recently, an RCT conducted in 146 individuals with a non-affective psychotic disorder (i.e. schizophrenia, schizoaffective disorder, or delusional disorder) demonstrated that a brief course (6-session) of manualized cognitive behavioral-based therapy targeting worry significantly reduced the severity of persecutory delusions relative to treatment as usual (TAU) (Freeman et al., 2015). This intervention (WIT) also reduced severity of worry, general psychotic symptoms, and increased personal well-being. All individuals in the trial had active persecutory delusions at the time of recruitment. While the efficacy of this intervention on reducing the severity of persecutory delusions has been demonstrated, whether reduction in delusion severity is related to changes in belief updating remain unknown.

In the proposed study, we aim to examine the impact of a previously established psychotherapeutic intervention for persecutory delusions on computational parameters of belief updating (e.g. volatility) and associated brain activation in individuals with psychotic disorders. Identifying whether belief updating ability changes in response to a therapeutic intervention would advance our understanding of delusions in the following ways: 1) determine whether changes in persecutory delusion severity are associated with changes in belief updating ability, clarifying mechanistic models of delusions; 2) identify neurobiological correlates of changes in persecutory delusion severity, providing future neurobiological targets for intervention; and 3) begin to examine whether psychotherapy is a mechanism-of-change for belief updating ability and persecutory delusions (i.e. whether changes in belief updating parameters mediate the relationship between treatment and change in delusion severity).

We propose an RCT in which individuals randomized to receive either WIT or Befriending therapy, an active control psychotherapy focused on the individual's interests. Inclusion of an active control group will extend previous findings against TAU and begin to address whether cognitive-behavioral interventions (versus the general factors of therapy) impact belief updating. We will recruit approximately 150 participants to enroll 60 adults (18-65 years) with a non-affective psychotic disorder (i.e. schizophrenia-spectrum disorder, delusional disorder). Participants will be randomly assigned to either the WIT(n=30) or Befriending (n=30) groups. The treatment lasts for 8 weeks. Both groups will be asked to complete an fMRI scan at baseline and end of treatment, as well as mid-treatment cognitive and clinical assessments and assessments at 24-week follow-up.

3.0 INCLUSION/EXCLUSION CRITERIA

Inclusion criteria

1. Men and women age 18 - 65.

2. Communicative in English.
3. Premorbid IQ >79 (WTAR)
4. Provide voluntary, written informed consent.
5. Physically healthy by medical history.
6. Weight <300 lbs
7. Stable medication regimen over at least the past two weeks, including the use of either an oral or intramuscular administration of an antipsychotic medication.
8. Diagnosis of a non-affective psychotic disorder (schizophrenia, schizoaffective disorder, schizophreniform disorder, delusional disorder, brief psychotic disorder, psychosis NOS) confirmed by Structured Clinical Interview for DSM-5 (SCID), diagnostic interview with a trained clinician, and/or review of electronic medical record.
9. A persecutory delusion scoring at least a 3 on the conviction scale of the Psychotic Symptoms Rating Scale (PSYRATS) that had persisted for at least two weeks and that was not considered the direct result of substance use
10. A clinically significant level of worry, as shown by a score of at least 44 on the Penn State Worry Questionnaire (PSWQ).

Exclusion criteria
1. Age less than 18 or greater than 65.
2. Not communicative in English.
3. Premorbid IQ < 79 (WTAR)
4. Unable to provide written informed consent.
5. Serious medical or neurological illness known to interfere with cognitive functioning (for example, HIV infection, any demyelinating disease such as Multiple Sclerosis, and active hepatitis).
6. History of severe head trauma with loss of consciousness >30 minutes.
7. Weight >300 lbs
8. Primary diagnosis of alcohol or substance use disorder or personality disorder
9. Subjects who are actively involved with individual cognitive therapy, engaging with therapy more frequently than twice per month (Past experience with individual therapy is not an exclusion)

Participants who have conditions that preclude MR scanning (as defined in the MRI Screening Form) will be excluded from the imaging portion of the study.

4.0 ENROLLMENT/RECRUITMENT

Study participants will be recruited from the following sources:

1) PGPR: Study participants will be recruited from the Psychiatric Genotype/Phenotype Repository (PGPR, IRB #080606): The PGPR study includes a clinical interview during which diagnostic and clinical history data is collected. At the end of this interview, individuals will be asked by one of the key research personnel listed on this application if they are interested in participating in the study. The PI of this study is a key study personnel for the PGPR study. Subjects will be read a script describing the study (StudyScript). Subjects will be told that the study is separate from their participation in the PGPR study and choosing not to participate in the study will not affect their participation in the PGPR study. In addition, individuals that have previously completed the PGPR study may be contacted and asked if they would like to participate in the study. The consent form for the PGPR study states that subjects may be contacted and asked if they would like to take part in future studies. In this case, prior PGPR participants will be contacted and told about the current study, and screened if they are interested (Phone Screening Script).

2) Vanderbilt Psychiatric Hospital and Adult Outpatient Clinic: Adult individuals who will likely meet criteria for one of the proposed study diagnoses (as determined by chart review, participation in another clinic study,

etc.) will be approached by someone familiar to them (e.g. a member of their care team) to see if they are willing to discuss participation in this study (see StudyScript). If the patient assents, their name will be given to a research staff member who will contact them to discuss the study in more detail, either in person (Screening Form) or on the phone (Phone Screening Script). Patients may also respond to advertisements, and in such cases, may be screened by phone (Phone Screening Script). If the subject meets criteria for the study, he or she may be scheduled for participation in the study.

3) SIRB: The General Factor of Psychopathology in Psychosis and Mental Illness (Comprehensive Survey of Psychological Symptoms): Study participants will be recruited from the SIRB Study (SIRB, IRB #191321): The SIRB study includes a clinical interview during which diagnostic and clinical history data is collected. At the end of this interview, individuals will be asked by one of the key research personnel listed on this application if they are interested in participating in the study. The PI of this study is a key study personnel for the SIRB study. Subjects will be read a script describing the study (StudyScript). Subjects will be told that the study is separate from their participation in the SIRB study and choosing not to participate in the study will not affect their participation in the SIRB study. In addition, individuals that have previously completed the SIRB study may be contacted and asked if they would like to participate in the study. The consent form for the SIRB study states that subjects may be contacted and asked if they would like to take part in future studies. In this case, prior SIRB participants will be contacted and told about the current study, and screened if they are interested (Phone Screening Script)

4) Nashville Community: Although Vanderbilt provides an excellent resource for recruitment, there are many individuals getting services at other locations (or who are not in services) who may benefit from or be interested in the research study. Therefore, we plan to place flyers (Flyer or Patient_Flyer_8.23) in mental health clinics in Nashville (e.g. Mental Health Co-operative, Centerstone) if allowed to by the facilities. We may also speak with individuals at NAMI meetings or advertise the study to outside providers, using the IRB-approved flyers.

5) Negative Valence Systems in Schizophrenia: Study participants will be recruited from the Negative Valence Systems in Schizophrenia (IRB# 211116): The study includes a clinical interview and self-report questionnaires where diagnostic and current symptomatology is collected. At the end of this study, individuals will be asked by one of the key research personnel listed on this application if they are interested in participating in the study. The PI is also included as a key research personnel for the Negative Valence Systems in Schizophrenia study. If a participant is there for the Negative Valence study in person, subjects will be read a script describing the study (See StudyScript). Subjects will be told that the study is separate from their participation in the Negative Valence study and choosing not to participate in the study will not affect their participation in the Negative Valence study. If they are interested, follow-up screening will occur (see ScreeningForm). In addition, individuals that have previously completed the Negative Valence study may be contacted and asked if they would like to participate in the study.

All study procedures is expected to take place at the Vanderbilt Psychiatric Hospital or in the Vanderbilt Institute of Imaging Science. If, however, the current environment and VUMC regulations prohibit or limit in-person study visits (due to coronavirus restrictions), cognitive and clinical assessments, as well as CBTp intervention study visits, may take place over Zoom. Administration of psychotherapy through telehealth is now well-established at VUMC and Zoom therapy sessions would be conducted with the consent of study participants. The PI, Dr. Julia Sheffield, is a licensed clinical psychologist and an outpatient psychotherapist within the VUMC Department of Psychiatry and Behavioral Sciences. She has been conducting weekly individual and group CBTp therapy over Zoom telehealth since 03/2020. We will recruit adult men and women with psychosis, age 18-65. All participants will provide written informed consent. This study will occur over an 8-week period.

Inclusion of women and minorities: We will make every effort to recruit equal numbers of men and women. We will not exclude subjects based on gender or minority status. We minimize coercion throughout the study by repeatedly informing subjects that they may discontinue study procedures at any point, or opt-out of any specific part of the study they become uncomfortable with completing.

Planned Enrollment: We plan to enroll up to 150 subjects, with the goal of randomizing 30 individuals to each therapy group.

Monetary Compensation: Study participants will be paid $15/hour for the clinical and behavioral portions of each visit and $80 for the 1-hour MRI scan. If a diagnostic interview is conducted, participants will receive an additional $15/hour at visit 1. Participant will not be compensated for attending intervention visits, however their transportation may be funded if they are unable to attend study visits without this assistance. If, following informed consent, a participant does not meet criteria based on symptom severity and/or diagnosis, they will be compensated $25 for their time. Subjects withdrawing consent during the MRI Scanning session will receive $10 plus $10/hour for testing outside the scanner, up to a maximum of $20. Any extra time spent in the study resulting from a technical problem with the MRI scanner will receive $15/hour. The same pay schedule will be used for all 4 study visits. Visit 1 will include the clinical assessments/self-report questionnaires/Cognitive tasks, MRI scan, and a possible diagnostic interview so participants will be paid up to $200 Visit 2 will include only the clinical assessments/self-report questionnaires/Cognitive tasks so participants will be payed $15/hour Visit 3 will include the clinical assessments/self-report questionnaires/Cognitive tasks and MRI scan so participants will be payed up to $155 Visit 4 will include only the clinical assessments/self-report questionnaires/Cognitive tasks so participants will be payed $15/hour Including all visits, participants will be payed a total of approximately $425.

5.0 STUDY PROCEDURES

This study has more than one part. We plan to complete these parts during either the same or different study visits. The different parts of the study are detailed below and include: answering questions about mental health symptoms, completing questionnaires, completing tasks on the computer, getting an MRI, and engaging in weekly 50-minute psychotherapy sessions over the course of 8-weeks. A single study visit is expected to last between 2 to 5 hours.

1. Screening and consent
2. Diagnostic Interview
3. Clinical Symptom Assessment
4. Cognitive Task Administration
5. Self-Report Rating Scales
6. MRI Scanning
7. Randomization
8. Treatment
9. Mid-treatment assessment (4-week)
10. End-of-treatment assessment (8-week)
11. Follow-up assessment

5.1 Screening and Consent: Subjects will be asked if they would like to participate in the current study. This screening will occur over the phone or in person using the Screening Form or Phone Screening Script. Informed consent will be obtained by study personnel directly involved in the research (i.e. research staff or the PI). Personnel have completed IRB training and have considerable experience running studies on psychiatric populations. Informed consent will be obtained in the research offices at the Psychiatric Neuroimaging

Program or over Zoom. A research staff member will explain the applicable procedures and the possible risks and benefits to the subjects. The details of the informed consent procedure are as follows:

1. The investigator or research staff and the subject will read together the entire consent form.
2. The subject will be asked details about the study. To document that the subject has read the consent form and has the capacity to understand the most important details, the investigator will use the appropriate Informed Consent Survey. The questions will be read by the investigator or research staff and the answers will be recorded. If the subject is unable to answer any of the questions, or if the subject demonstrates a lack of understanding, the investigator or research staff member will then review the details of the study again. Subjects who are unable to answer the questions, even after additional information is provided, will be excluded from the study.
3. The subject will be informed that regardless of whether or not they take part in this research study, it will not affect their treatment, payment or enrollment in any health plans or affect their ability to get benefits or care in any way.
4. The subject will be provided with copies of the signed consent form and the Informed Consent Survey (including the answers given) at the time of the initial visit.

It will be emphasized to all subjects that their participation is completely voluntary, and that even after signing the consent document they are still free to withdraw from the study at any time; in which case they will be compensated for the portion(s) of the protocol they did complete.

5.2 Diagnostic Assessment: Under the supervision of the PI, a trained research assistant will administer the Structured Clinical Interview for DSM-V (SCID), a clinician-rated assessment of psychiatric disorder. Diagnoses are made both for current and lifetime periods. The SCID is reliable and valid in psychiatric populations (significantly superior to standard clinical interview). We will supplement the SCID with the psychotic disorders module from the Diagnostic Interview for Genetics Studies (DIGS), which provides more information for the accurate differential diagnosis of psychotic disorders. Clinical Assessment and Cognitive Tasks: Almost all of the clinical and cognitive tests have been used in our past studies. They are not burdensome and we allow breaks. All scales will be completed before or on the day of the neuroimaging procedures. All patients are asked to review their substance use and current medications at every visit.

As stated previously, subjects who were recently recruited in other psychiatry studies (PGPR, g-factor) may not be required to participate in components of this study that would duplicate efforts (e.g. SCID interview). We plan to include data from those studies in the analyses of data from this study. If an excessive time has passed since an individual participated in a PGPR study, portions of the diagnostic interview may be re-administered to determine if there have been any interval changes in a patient's diagnostic status. Healthy controls may also undergo the SCID. Additionally, subjects may not be required to participate in a SCID interview if their diagnosis may be confirmed by review of their electronic medical record.

5.3 Clinical Symptom Assessment: Positive and Negative Syndrome Scale: : a 30-item clinician-rated scale designed to rate both presence of active psychotic symptoms and of cognitive symptoms, like alogia, anhedonia, and flattened affect (Kay et al., 1987). This measure has fair to good psychometrics (Peralta and Cuesta, 1994) and takes about 30 minutes to complete. Psychotic Symptom Rating Scale (PSYRATS): The PSYRATS is a clinician-rated scale designed to measure the level of preoccupation, conviction, distress, and functional impairment as a result of active delusions and hallucinations. The PSYRATS is completed by trained interviewer and is expected to take about 20 minutes to complete**.** Quality of Life Scale: (Heinrichs et. al. 1984) is a 21-item clinician-rated measure used to rate current functioning in several areas of life for psychotic patients including interpersonal relationships, occupational roles, and richness of personal experience. The QOLS takes approximately 5-10 minutes to complete. Personal and Social Performance Scale: (PSP; Nasrallah et al, 2008) is a 4-subscale clinician-rated instrument designed to evaluate the severity of functioning

in the domains of socially useful activities, personal and social relationships, selfcare, and disturbing and aggressive behaviors. It has good reliability and validity and takes about 10 minutes to complete. Time Use Survey is a 15-20 minute survey that assesses activity (leisure, work/school, child care, house work/chores).

5.4 Cognitive Task Administration

Wechsler Test of Adult Reading (WTAR): (Harcourt assessment, 2001) The WTAR is a standardized test of reading that requires subjects to read a list of words aloud as best as they can. The WTAR is highly correlated with overall intellectual abilities and provides a quick measure of intellectual functioning, especially pre-morbid functioning in the case of patients. The WTAR takes approximately 5 minutes to administer.

Screen of Cognitive Impairment in Psychiatry (SCIP): : used to estimate cognitive function in individuals with psychiatric illness. This assessment is a valid and reliable tool (Purdon, 2005) and takes 10-15 minutes to administer.

Belief Against Disconfirmatory Evidence (BADE) Task: You will be presented with a series of statements about different fictional individuals. You will then rate the plausibility of four explanations for each scenario. This task takes approximately 20-30 minutes.

3-Probabilistic Reversal Learning Task: In this task, we will show you three decks of cards on the computer screen. Each deck will have a hidden probability of reward. Using the keyboard, you will select the deck of cards you think will earn you the greatest number of points. You will then receive feedback about the reward outcome for the deck you selected (+100 or -50 points). You are instructed to find the best deck, that will earn you the most points. However the best deck may change throughout the experiment. This task takes approximately 15-30 minutes to complete.

Levels of Emotional Awareness Test (eLEAS): In this task, participants will be read up to 20 scenarios describing social interactions. After each scenario they will be asked two follow-up questions about how the scenario may make someone feel. This task takes approximately 15 to 30 minutes.

Horizon Task: In this task, participants will choose between two slot machines, like what you might find in a casino. Each machine will reward them in points, between 1 and 100. Participants may stick with the same machine or switch between them. The goal is to earn as many points as possible. This task takes approximately 12-15 minutes.

Emotional Stroop - Participants will observe neutral and negative words that are presented in different colors. They will be asked to state the color the word is printed in. This task takes approximately 15-20 minutes to complete.

Attentional Blink Task - Participants will observe a series of neutral images and asked to identify when they see a specific target image by pressing a button. Sometimes, a more threatening image will appear on the screen, however their task will not change. This task will take 15-20 minutes.

Anticipating Future Outcomes - Participants will be presented with different possible future events (e.g. having an argument with a friend) and will be asked to rate how likely that is to occur and how bad it would be for them if it did occur. This task will take about 10 minutes.

Clinical Assessments Diagnostic Interview (Visit 1) You may be asked to complete an interview about psychiatric symptoms you currently have or have experienced in the past. These will include questions about

your mood, anxiety, substance use, psychosis, and any compulsive behaviors. You will be asked about when these experiences happened and how they impacted your daily life. This interview is expected to take 1-3 hours and you will be offered breaks as needed.

5.5 Self-Report

Self Esteem Scale: is a 40 item self-reported questionnaire designed to measure the self and perceived self-esteem. The Self Esteem Scale takes approximately 5 minutes to complete.

Baer Grant Defeatist Beliefs Scale: (Grant & Beck 2008) is a 5 item self-report questionnaire designed to measure one's defeatist beliefs about themselves. This scale should take less than 5 minutes to complete

Baer Grant Asocial Beliefs Scale: (Grant & Beck 2010) is a 7 item yes-no self-report questionnaire asking about one's interaction with others. This scale should take less than 5 minutes to complete.

Beck Depression Inventory-II : (BDI-II, Beck et. al. 1996) is a 21-item self-reported questionnaire designed to measure the severity of depression in adolescents and adults and addresses all nine of the diagnostic criteria for a major depressive episode that are listed in the DSM-IV-TR. The BDI-II takes approximately 5-10 minutes to complete.

Penn State Worry Questionnaire: The PSWQ is a widely used measure of worry severity. It assesses worry through a 16-item self-report scale using a Likert Scale. The PSWQ take approximately 5 minutes to complete.

Dunn Worry Questionnaire: The Dunn Worry Questionnaire is a 15-question self-reported measure of worry over the past month using a Likert Scale. Worry questions focus on general worry as well as worry about others trying to upset or harm you. The DWQ takes approximately 5 minutes to complete.

Perseverative Thinking Questionnaire: This 15-question self-report assesses perseverative thinking style on a Likert Scale. The PTQ takes approximately 5 minutes to complete.

Warwick-Edinburgh Mental Wellbeing Scale (WEMWBS): (Brown et. al. 2007) is a 14 item self-report scale that covers both feeling and functioning aspects of mental wellbeing like resilience and protective factors. The WEMWBS is used in multiple geographical locations, languages, and cultural contexts with extensive validity. The WEMWBS takes approximately 5 minutes to complete.
Choice of Outcome in CBT for Psychosis (CHOICE): (Greenwood et. al. 2009) is a 24 item self-report scale that provides scores for the severity and the satisfaction of an individuals intervention with a range of difficulties. The measure has good test-retest reliability, face and construct validity, and sensitivity to change. The CHOICE takes approximately 5-10 minutes to complete.

Beck Cognitive Insight Scale: (BCIS; Beck et al. 2004) is a 15-item self-report questionnaire designed to evaluate patients' self-reflectiveness and their overconfidence in their interpretations of their experiences. The scale demonstrates good convergent, discriminant, and construct validity. The BCIS takes approximately 5 minutes to complete.

Difficulties in Emotion Regulation (DERS-16): This 16-item self-report assesses one's ability to regulate emotions when upset. It takes approximately 5 minutes to complete.

The Sleep Conditions Indicator (SCI) is an 8-item assessment of sleep quality and behaviors. It should take about 3

Working Alliance Inventory (WAI) is a 12-item questionnaire about how an individual feels about their therapist/counselor

Credibility/Expectancy Questionnaire is a 6-item questionnaire about expectations and perceived credibility of a therapy intervention.

Revised 28-Item Racial and Ethnic Microaggression Scale (R28REMS; Forrest-Bank et al., 2015): This revised version of Nadal (2011)'s 45 item Racial and Ethnic Microaggression scale includes 28 items assessing frequencies of covert discrimination events. This measure takes about 10 minutes to complete.

General Ethnic Discrimination Scale (GED; Landrine et al., 2006): an 18-item measure of perceived ethnic discrimination. This takes about 5-10 minutes to complete.

5.6 MRI Procedures (Baseline and Week 8): MRI scans of the brain will be obtained using the MRI scanners in the Center for Human Studies in the Vanderbilt University Institute for Imaging Science (VUIIS), located in the Vanderbilt Hospital and Medical Center North. This will require approximately 60-90 minutes and no contrast agent will be used. Participants may be invited to complete multiple MRI sessions as part of the study.

5.6.a MRI Screening: The participant will fill out the MRI Procedure Screening Form. The purpose of this form is to ensure that there are no implanted medical devices or metals that could injure the subject if exposed to a high magnetic field. This form will be filled out in a private room adjacent to the MRI scanner and reviewed by the MR Technologist and the research assistant.

5.6.b Scanning Procedures: Imaging studies will take place on a Phillips 3.0 T MRI scanner. The magnet and the magnet's control console are in separate rooms, but the investigator and the subject will be in voice communication at all times, and the investigator will be able to see the subject through a window. All data acquired on the scanner will be securely transferred over the Vanderbilt network to the Institute of Imaging Science's server. Imaging data will be de-identified and only be accessible to the PI, co-investigators, and VUIIS personnel. Scanning procedures may include: 1) Structural imaging, 2) Resting state functional imaging, 3) Functional imaging with task – while in the scanner, participants will complete one of two tasks:

1. **3-Probabilistic Reversal Learning Task:** In this task, we will show participants three decks of cards on the computer screen. Each deck will have a hidden probability of reward. Using the keyboard, they will select the deck of cards they think will earn them the greatest number of points. Participants will then receive feedback about the reward outcome for the deck they selected (+100 or -50 points). They are instructed to find the best deck, that will earn them the most points. However the best deck may change throughout the experiment. This task takes approximately 15-30 minutes to complete.

5.7 Randomization
We propose an RCT with two treatment groups: Worry Intervention Training (WIT) and Befriending Therapy (called Connection Therapy during clinical trial). To produce balanced baseline characteristics, a computer program will be used to implement random assignment to treatment groups. To produce balanced sample sizes, desirable for statistical power and resource management reasons, the software uses existing group sizes to adjust the probability of assignment to particular groups. The PI will conduct the randomization procedures following the baseline assessment and will only tell the relevant interventionist which group the participant was assigned to. This will conceal allocation from assessors and coders.

5.8 Treatment
After the initial study visit, subjects will be randomly assigned to receive one of two types of psychotherapy with a qualified psychotherapist: Worry Intervention Training (WIT) or Befriending therapy (also referred to as "Connection Therapy" in the ICD to minimize expectancy bias. In both therapy conditions, subjects will meet with a psychotherapist for 6, 50-minute sessions over the course of 8 weeks. In WIT, they will speak with the

therapist about the impact of worry on their daily life. The therapist will offer tools for managing those worries that they can practice at home, in order to free up their headspace to focus on things that are important to them. In Befriending Therapy, therapy sessions will focus on taking time off from thinking about life's difficulties by connecting with your therapist around topics that interest you. Both types of therapy have been shown in research trials to make people feel safer, happier, and more motivated to do the things they want to do.

5.9 Mid-Treatment Assessment
In the middle of treatment (Week 4) study participants will be asked to complete a brief, 2-3 hour assessment. This assessment will include clinical interview, self-reports, and the 3PRL task (described above).

5.10 End of Treatment Assessment
At the end of treatment (Week 8), all assessments (except the diagnostic interview and childhood trauma questionnaire), as well as the MRI, will be repeated.

5.11 24-Week Follow-up
At week 24, research subjects will be invited to complete a follow-up visit. This visit will be 2-3 hours and focus on clinical interview, self-report, and select cognitive tasks

6.0 RISKS

The following procedures may have risks: clinical assessments, neuropsychological and cognitive testing, MRI scanning, psychotherapy intervention.
Confidentiality: The risk of confidentiality will be minimized by thorough training of all research personnel and the high level of security in our information systems. All subjects will be assigned a Subject ID numerical code to maintain anonymity of the participants and their test results. The Subject ID will serve as the primary identifying information on research data forms. Research personnel will comply with the ethical standards set forth by the IRB, NIH and the American Psychological Association. The records of this study, to include paper records and electronic records, will be kept private and secured in locked filing cabinets or in password-protected databases (RedCap) and folders (for videos). In any published report, we will not include any information that will make it possible to identify a subject.

If at any point during the study sessions the participant expresses any intent to harm themselves or others or a researcher becomes concerned for their safety, the researcher will contact the primary investigator or another licensed clinical psychologist on the research team. The clinical psychologist will conduct the brief ASQ suicide risk screen and the Columbia suicide severity scale for the past month. If the clinician is concerned for the participant's immediate safety they will continue with a safety plan and provide mental health resources and discuss alerting a family member or loved one of the concerns. If the participant cannot contract for safety and the clinical psychologist believes they may be a threat to themselves or others then the participant would be brought to the Psychiatric Assessment Services (PAS) for an evaluation for inpatient admission. If the study visit is occurring over zoom and an individual cannot contract for safety, the clinical psychologist will call 911 to the individual's address. Participants will be informed of this procedure during the informed consent process and again at the start of therapy.

Clinical Assessments: The risk of the clinical assessments involves the possibility that participants might be asked questions that cause them distress. These would be questions consistent with any diagnostic and clinical evaluation. In order to address this risk, participants will be told they can decline to answer any questions and can stop the evaluation at any time. Participants may also feel fatigued, bored or frustrated by the length of the clinical assessments. Participants will be allowed to take breaks at any time and assessments can be completed across multiple sessions if needed.

Cognitive Testing: The risk of neuropsychological and cognitive testing involves the possibility that participants may experience mental fatigue or anxiety. The nature of the tests and that the data are for research purposes only will be described to study participants. Any neuropsychological tests that elicit excessive anxiety will be skipped. Subjects will be informed beforehand that they may take a break at any point during administration of neuropsychological tests and cognitive paradigms.

MRI: With respect to MRI scanning, there are no known health risks associated with the magnetic field produced by 3.0T scanners in healthy subjects. The FDA has indicated that they consider MR imaging on machines up to 4.0T to pose no risk. Activation studies with fMRI are routine, and are in place in a number of institutions in the U.S. and around the world. There may be minor discomforts for subjects because they must lie motionless for an extended period of time. Physical discomfort will be minimized with cushions and foam used to hold the head without placing undue pressure on it. Subjects are allowed to dress comfortably, and they are covered with a light cotton cover to keep them warm. Participants must wear hearing protection. Persons at primary risk from exposure to the magnet include persons with metal surgical implants, and anyone with implanted electric/magnetic devices (e.g. a pacemaker, orthodontic braces, aneurism clips, IUDs). Such individuals will be excluded from the imaging portion of the study. People at secondary risk will also be excluded from the imaging portion of the study. These include people with significant chronic physical illnesses (e.g. asthma, heart conditions, diabetes, hemophilia) or neurological disorders (e.g. epilepsy, autism, or other syndromes). To ensure that no such subjects in these risk groups are included in the study, subjects must fill out a screening that is reviewed by the MRI operator prior to scanning (see attached documents). There is a risk that some subjects may be anxious (claustrophobic) about lying in an enclosed space. We anticipate that the occurrence of such responses will be minimal given that subjects are told about the MRI scanning procedures and that they will be placed in a hollow tube-like scanner beforehand during the consent process. Moreover, subjects will be able to stop the session at any time, by pressing a switch held in one hand. The switch activates a loud buzzer in the control room. There is also an intercom voice link with the control room that allows verbal communication between subject and experimenters. All study personnel who have contact with participants in the scanner are trained in the above described risks and procedures to avoid risk. In the highly unlikely event of an adverse event occurring during scanning, a Radiology resident is on-call at all times to render assistance if necessary. This person can be reached by calling the Medical Center Operator at 322-5000 and asking them to page the Radiology resident on call (Beeper #0123).

As a consequence of the scanning procedure an abnormality might be detected in a subject's brain. In the event that the MRI technologist suspects there may be an abnormality, we will have the technician on duty burn a CD containing DICOM images of the subject. The RA running that participant through the study will take that disc to the radiology department in Medical Center North and leave it with Dr. Taylor Davis's assistant. Dr. Davis is a neuroradiologist who has agreed to look at our incidental findings. Dr. Sheffield will send Dr. Davis an email to confirm the disc was received, and Dr. Davis will confirm any findings. If the neuroradiologist finds the abnormality requires attention, the PI will contact the subjects via phone as soon as possible. On the phone call we will provide the subject with this information, and help them identify referrals as needed.(see the Communicating_Incidental_Findings document for a template of a phone call)

Intervention: The intervention will be conducted in a safe, supportive clinical environment implemented by trained psychotherapists employed at Vanderbilt University Medical Center. Intervention procedures (i.e. the delivery of WIT or Befriending therapy) will mirror procedures regularly conducted in the Vanderbilt Adult Psychiatry Outpatient Clinic (VAPOC), including review of confidentiality and its limits and the use of either Zoom teletherapy or private, dedicated clinical offices. Notes from the intervention sessions will not be included in the participant's medical record and will not be directly shared with their other providers except in the case of imminent safety risk. Notes about the therapy sessions will be anonymized and kept in encrypted files on a password-protected server. Video recordings will also be kept on password protected servers that is only accessible to the research staff and will not include any identifying information.

The intervention, particularly the WIT intervention, is designed to address distress and preoccupation surrounding persecutory delusions. Discussion of these topics may be upsetting to some participants. However, all therapists in the trial will have at least 3 years of experience working with individuals with psychotic disorders and will be fully trained in the WIT intervention by experts in the field. The PI is a licensed clinical psychologist with over a decade of experience working with individuals with psychosis, and the PI will be providing supervision and support for all therapists throughout the trial, in order to help manage issues that arise during therapy. Furthermore, during the screening process, participants will be informed that one of the therapies is designed to address feelings of worry about others harming them, and that these topics will be addressed in therapy. They will be reminded throughout the screening and consent process that the research study includes therapy sessions with a professional therapist, and will be asked whether this is something they want to engage in. Distress during or between sessions will be monitored by the participant's study therapist and the intervention will be adjusted to minimize distress. For instance, if a participant is distressed by conversation about a specific topic, the therapist will discuss with the participant if they would like to change the topic and may conduct a grounding or breathing exercise to reduce distress in session. Therapists may check in with participants throughout the week depending on the participant's preference.

Befriending therapy involves discussion of neutral topics that interest the participant, such as sports or music and is specifically not focused on the individual's illness or symptoms. Befriending therapy allows for a development of a therapeutic alliance, and therefore some individuals experience loss of that support after therapy ends. The therapist will work with each participant to address this potential loss and will refer them to additional therapy services after the 8 weeks, if requested.

Reporting of Adverse Events (AEs) or Unanticipated Problems involving Risk to Participants or Others

The PI and study personnel will review any AEs that occurred during a participant's study experience and determine their cause and severity. AEs rated as grade two or higher on the scale below will be reported to the IRB within 7 days of the receipt by the Investigator when the report identifies a new risk or a change in the risk-potential benefits profile. In conjunction with the IRB, the PI then will determine if modifications to the protocol are warranted. At the end of each year that this study continues, an annual report of AEs will be collected and shared with the IRB.

Grading of Severity of Adverse Events (AEs)
0: No AE or within normal limits.
1: Mild AE.
2: Moderate AE.
3: Severe AE resulting in inpatient hospitalization, a persistent or significant disability/incapacity, or serious injury.
4: Life-threatening or disabling AE.
5: Fatal AE.

During weekly lab meeting, Dr. Sheffield will review data collection procedures with study staff and discuss any data collection problems which have been encountered. Quality control analyses will be conducted throughout the recruitment phase and will be discussed with study staff during lab meetings in an effort to quickly identify issues with data collection accuracy.

All subjects will be monitored by study staff throughout the study for any AEs or other safety issues. This study does not involve any known health risks. However, subjects may feel upset or uncomfortable by study procedures, such as the evaluation of clinical symptoms or the MRI scan. Subjects will be told that they may discontinue the experiment at any time they feel upset or uncomfortable. Study staff will immediately inform Dr. Sheffield of any subjects who becomes upset or withdraws themselves during the study, or experiences any adverse event.

If at some point during the study research staff may become concerned about a study participants' safety, they will follow the Crisis Assessment Procedures described below. Examples of concerning behavior include statements from the participant that life may not be worth living, that they would prefer to be dead, or that they have been having suicidal thoughts in the past month. These statements may occur in the context of the clinical or diagnostic interviews, but will also be monitored throughout the study visit by research staff, as they may occur unexpectedly. If such statements are made, a licensed mental health professional (the study PI, a licensed clinical psychologist, or licensed study staff) will assess risk as described below.

Crisis Assessment Procedures

1. Express concern for subject. Point to specific behavior of concern:

E.g. You said that you think a lot about killing yourself and this concerns me...

E.g. You said that you wish you were not living and this concerns me...

2. Ask subject to speak with a licensed psychologist or psychiatrist

E.g. I want to make sure you are safe. Would you talk with the study psychologist for a few minutes before you leave?

a. If subject is willing, find study PI (a licensed psychologist) or a study collaborator who is a licensed psychiatrist or psychologist.
b. If a study PI or collaborator is not available, ask subject to walk to Psychiatric Assessment Services (1st floor of VPH) to talk to on-call doctor.

3. If the subject is willing to speak with the study PI or collaborator, a suicide screen will be conducted (Suicide Screen NIMH).

a. If the subject answers "yes" to any questions on the screen, a more thorough assessment will be conducted by the licensed professional (Suicide Risk Assessment C-SSRS), focused on the past month. If risk appears imminent, a discussion will be had with the subject about going to the Psychiatric Assessment Services (PAS). If the subject is unwilling to go voluntarily, VUPD 11911 will be called to assist. If this occurs during an online study visit, then 911 will be called if the subject is unwilling to take safety measures (e.g. going to PAS themselves, calling their mental health provider).
b. If the subject answers "no" to all questions, the licensed professional will provide them with a list of resources (Mental Health Resources; Behavioral Safety Net) and discuss how they can maintain safety (E.g. I am still somewhat concerned about your safety, is there a family member you live with who may be able to keep an eye on you over the next few days? would you be willing to give your psychiatrist a call to let them know how you have been feeling?)

4. If subject is not willing to speak with study PI, ask subject if there is a family member who could pick them up from the appointment. Re-express concern for the subject's safety.

E.g. I'm very sorry that the interview upset you. It may be difficult for you to concentrate on driving now. Is there a family member I could call to drive you home?

• If subject has made directly threatening statements toward self or others, do not let subject leave-even if with family member. Tell family member that you are concerned for subject's safety and that they should talk to a doctor before they leave. If subject leaves anyway, call VUPD 11911

4. If subject refuses all offers (and has not made directly threatening statements or gestures):
a. Walk subject out of building. If subject smokes, offer to sit with subject while they smoke and continue interview. If subject calms while smoking, repeat steps 2 and 3.
b. If subject does not smoke, offer to sit with subject in an open-air area and continue interview. If subject calms while outside, repeat steps 2 and 3.
c. If subject continues to refuse help, advise subject to call regular therapist or psychiatrist as soon as possible.
d. If subject has made directly threatening statements regarding self or others, note subject's vehicle and clothing. **Call VUPD 11911**

CRITICAL RISK FACTORS
-subject has a specific plan or has implied a plan
-subject has access or has implied access to weapon
-recent attempt or history of attempts
-subject dwells on death or has strong suicidal ideation

5. Make thorough notes describing concerning behavior, help offered, and subject's responses. Place in subject file.

6. Debrief study PI as soon as possible. PI will notify the IRB within 7 days if AE is found to be a 2 or higher on the scale above.

7.0 Study Withdrawal/Discontinuation

Subjects may elect to withdraw from the study at any point. If this occurs due to adverse effects, immediate treatment will be provided. Subjects may be discontinued by the investigator for a number of reasons. These include the following:

1. mental fatigue or anxiety to the point that it is interfering with their ability to complete the study
2. significant distress during the structured, manualized therapy sessions, such that the therapy does not appear clinically appropriate. Withdrawal will not affect any future clinical care at Vanderbilt University Medical Center.
3. Subjects who have adverse events of moderate severity or greater who, in the judgment of the principal investigator, may be unable to complete the study or whose health may be compromised by further participation.

8.0 Statistical Considerations

Given that this is a clinical trial, following randomization, all participants who completed the baseline assessment and were randomized will be included in an intent-to-treat analysis, to capture as much data as possible. Regarding the primary aims, **AIM1**: linear regression will be used to test if the severity of persecutory delusions is associated with volatility at baseline, and whether change in delusion severity over 8-weeks is associated with change in volatility across all psychosis participants. These changes are expected to be stronger in the WIT group than in those receiving Befriending. **Aim 2**: Neurobiological underpinnings of volatility will be assessed pre/post treatment in psychotic disorder patients using task-based fMRI. It is hypothesized that activation in the ventral striatum, insula and dlPFC will covary with volatility during a belief updating task. Persecutory delusion severity is expected to be positively associated with volatility-related task activation. Patients receiving WIT are expected to demonstrate normalization (i.e. reduced) activation in these regions post-treatment, which will be associated with delusion severity, representing neurobiological targets for future treatment trials.

9.0 Privacy/Confidentiality Issues

All efforts, within reason, will be made to keep your personal information in your research record confidential but total confidentiality cannot be guaranteed.

All paper records will be kept in a locked filing cabinet. Electronic records will be stored in a passwordprotected database that only research staff has access to. Subjects will be assigned a numeric code that will be used to label all research data, including brain imaging scans. Only Dr. Sheffield and approved research staff will have access to this data, and will be able to connect the numeric codes with identifying information.
The 3-PRL task will be run through a secured, HIPPA-compliant server hosted by Yale University. Only de-identified data will be stored on this server. Only the PI and approved research staff will have access to any identifiable information associated with this data.

Clinical interviews may be recorded to allow for assessment of inter-rater reliability measurement between different study raters. Therapeutic sessions may be video recorded to ensure that the therapists are providing the therapy to the highest standard. These videos will be password protected and seen only by researchers involved with the study. If conducted over video-conferencing, access links to the video-call will be provided only to the research participant and approved staff. Video-calls will take place in private locations where risk of someone hearing or seeing the research visit is minimized. We are required by state law to immediately report any evidence of possible child abuse with identification of the alleged offender. If any information is revealed during this study concerning suicide, homicide, child abuse, or neglect, it is required by law that this be reported to the proper authorities. If at any point during the study sessions or psychotherapy sessions you express an intent to harm yourself or others or a researcher becomes concerned for your safety, the primary investigator or another licensed mental health professional on the research team will speak with you about these concerns and discuss a safety plan with you. If safety cannot be guaranteed and/or planned for, and the mental health professional believes you may be a threat to yourself or others, then your confidentiality may be breached, in line with legal standards. If information from the study is published or presented at scientific meetings, your name and other personal information will not be used. The Vanderbilt Institutional Review Board has the authority to review your research and medical records. This study may have some support from the National Institutes of Health (NIH). If so, your study information is protected by a Certificate of Confidentiality. This Certificate allows us, in some cases, to refuse to give out your information even if requested using legal means. It does not protect information that we have to report by law, such as child abuse or some infectious diseases. The Certificate does not prevent us from disclosing your information if we learn of possible harm to you or others, or if you need medical help. Disclosures that you consent to in this document are not protected. This includes putting research data in the medical record or sharing research data for this study or future research. Disclosures that you make yourself are also not protected.

All efforts, within reason, will be made to keep your protected health information (PHI) private. PHI is your health information that is, or has been, gathered or kept by Vanderbilt as a result of your healthcare. This includes data gathered for research studies that can be traced back to you. Using or sharing (“disclosure”) such data must follow federal privacy rules. By signing the consent for this study, you are agreeing (“authorization”) to the uses and likely sharing of your PHI. If you decide to be in this research study, you are also agreeing to let the study team use and share your PHI as described below. As part of the study, Dr. Sheffield and her study team may share the results of your study and/or nonstudy linked MRI scans, as well as parts of your medical record, to the groups named below. These groups may include people from the Federal Government Office for Human Research Protections and the Vanderbilt University Institutional Review Board. Federal privacy rules may not apply to these groups; they have their own rules and codes to assure that all efforts, within reason, will be made to keep your PHI private.

10. Follow-up and record retention

The study results will be kept in your research record for at least six years after the study is finished. At that time, the research data that has not been put in your medical record will be kept for an unknown length of time. Any research data that has been put into your medical record will also be kept for an unknown length of time.

Unless told otherwise, your consent to use or share your PHI does not expire. If you change your mind, we ask that you contact Dr. Sheffield in writing and let her know that you withdraw your consent. Her mailing address is 1601 23rd Ave. S., Suite 3057, Nashville TN, 37212. At that time, we will stop getting any more data about you. But, the health data we stored before you withdrew your consent may still be used for reporting and research quality.
