# Supplementary material for: Prior Expectations of Volatility Following Psychotherapy for Delusions: A Randomized Clinical Trial
Source: JAMA Netw Open. 2025 Jun 24;8(6):e2517132. doi: 10.1001/jamanetworkopen.2025.17132 (PMC12188364; doi:10.1001/jamanetworkopen.2025.17132)
Supplement: Supplement 2. — eMethods eResults eTable 1. Outcomes eTable 2. MRI Cohort eTable 3. Significant Clusters in Whole Brain Analysis eFigure 1. A) Task, B) Decision and Feedback-Periods Defined for fMRI Analysis, C) HGF Model eFigure 2. Parameter Recovery eFigure 3. Masks Used eFigure 4. Healthy Comparison Group [file jamanetwopen-e2517132-s002.pdf]

Supplemental Online Content

Sheffield JM, Sloan AF, Corlett PR, et al. Prior expectations of volatility following psychotherapy: a randomized clinical trial. *JAMA Netw Open*. 2025;8(6):e2517132. doi:10.1001/jamanetworkopen.2025.17132

eMethods
eResults
eTable 1. Outcomes
eTable 2. MRI Cohort
eTable 3. Significant Clusters in Whole Brain Analysis
eFigure 1. A) Task, B) Decision and Feedback-Periods Defined for fMRI Analysis, C) HGF Model
eFigure 2. Parameter Recovery
eFigure 3. Masks Used
eFigure 4. Healthy Comparison Group

This supplemental material has been provided by the authors to give readers additional information about their work.

© 2025 Sheffield JM et al. *JAMA Network Open*

eMethods

Participants
Participants were compensated $15/hour for the assessment battery plus $80 for a 1-hour MRI. Participants were not compensated for attending therapy sessions but were provided transportation or reimbursement for gas if needed. In addition to pre/post treatment, mid-treatment (4-week) and follow-up assessments (24-week) were also conducted, but did not include fMRI. Therefore, only data from the baseline and end-of-treatment timepoints are presented here to capture our primary pre-registered analyses.

Interventions
The CBTp intervention, developed by Freeman and colleagues, treats persecutory delusions through targeting worry-reduction in 6-sessions over the course of 8-weeks. It has demonstrated efficacy in reducing persecutory delusion severity in individuals with schizophrenia-spectrum disorders and persistent persecutory delusions with >50% conviction, as compared with standard care. This intervention is based on five modules that focus on: psychoeducation about worry, identifying positive and negative beliefs about worry, identifying triggers for worry, using worry periods, postponing worry using a series of techniques (e.g. activity, imagery, stress reduction) and increasing safe imagery usage. A "worry cycle" was developed collaboratively with the participant and therapist to outline how worry perpetuates feelings of being under threat, contributing to persecutory beliefs. When the participant agreed, the study therapist would contact them at least one time between sessions, via phone call or text, to check in on their use of intervention tools and offer support or guidance on the intervention. Individual sessions were conducted in-person for approximately 50-minutes.

Befriending therapy is a manualized intervention that incorporates non-specific but important aspects of psychotherapy, including time-in session with a therapist, positive expectancy, and non-judgmental positive regard[33]. Befriending involves a series of conversations that are friendly and social, but avoid active interventions of CBT, such as problem-solving, increasing awareness of thoughts and feelings, behavior planning, and drawing conclusions. Participants in this group were told the therapy was intended to give them "a break" from their worries and distressing thoughts to focus on everyday topics.

Task and Computational Modeling
Participants observed three decks of cards on a computer screen and were instructed to find the "best deck" and then stick with it, to gain the most points. They were told that all card decks contained winning (+100) and losing (-50) cards, but that one of the decks won the most often. Volatility was imbedded in the task in two ways: (1) participants were told that at some point during the task, the winning deck might change (reversal) at which point they should find the new "best deck"; (2) unbeknownst to the participant, halfway through the task, the reward contingencies of the decks changed. The first two blocks (80 trials) had reward contingencies of 90-50-10 and the final two blocks (80 trials) had reward contingencies of 80-40-20. Because none of the decks won 100% of the time, participants

needed to figure out if an outcome was probabilistic (random) or indicated a change in the environment (volatility). The task was conducted using E-Prime 3.0 software (Psychology Software Tools, Pittsburgh, PA) and took approximately 24 minutes to complete.

Behavior on the task was then modeled using a Hierarchical Gaussian Filter (HGF) as previously reported[16–18,34,35]. The HGF includes a 3-layer perceptual model and a response model. The highest level (Level 3) captures beliefs about the task environment, including how much it is changing over time (volatility). Prior beliefs about environmental volatility are captured in μ_3^0, reflecting how much the participant expects the environment to change; ω_3 captures how quickly participants learn about the task's volatility (meta-volatility learning rate); κ reflects how much beliefs about task volatility influence perceptions of the underlying reward probabilities of the decks (sensitivity to volatility or unexpected uncertainty). Parameter recovery was conducted based on simulated data and volatility priors were able to be recovered (p=.04) (Figure S2). The task and modeling are described in detail elsewhere[17] and analysis scripts are available at https://github.com/JuliaSheffield/CogMech_delusions.

Outcomes

The primary clinical outcome was the total score of the Psychotic Symptoms Rating Scale (PSYRATS)[37] delusions subscale (range, 0-16). During the initial assessment, a delusion statement was developed in collaboration with the participant. This statement described the primary persecutory delusion the person was experiencing with >50% conviction. The PSYRATS includes assessment of delusion conviction, distress, preoccupation, and disruption to life. The same delusion statement was rated throughout the trial, at subsequent assessments.

Our secondary clinical outcome was the positive subscale from the positive and negative syndrome scale (PANSS)[41], as this captures a broader picture of psychosis symptom severity. Sensitivity analysis was conducted on PANSS negative and general symptoms and depression from the Beck Depression Inventory (BDI)[42], which were not expected to relate to volatility beliefs, despite relationships with other computationally-derived reward-processing abnormalities[43,44].

Healthy Comparison Cohort

Healthy comparison participants (N=27) without a psychotic disorder diagnosis were recruited at VUMC from March 28, 2022 and May 28, 2024 and included for sensitivity analysis. Individuals provided written informed consent to take part in a longitudinal study at VUMC (IRB# 202462). In the context of this study, the 3PRL task was collected during acquisition of fMRI data at two timepoints, 8-weeks apart. Individuals were included in this cohort if they met the following criteria: between 18-55 years old, no history of traumatic brain injury (TBI) or serious neurological disorder, no current psychiatric disorder (past anxiety disorder and mild depressive episode allowed), not currently taking psychotropic medications, no current substance use disorder, no first degree relative with a psychotic

disorder, MRI-eligible. Data analysis for this cohort followed the same procedures as for the psychosis cohort.

Neuroimaging Data Collection
High resolution T1-weighted structural scans and four, six-minute functional MRI (fMRI) scans were collected at each study visit, for individuals eligible to complete the MRI protocol. Imaging data were acquired on 2 identical 3T Philips Intera Achieva MRI scanners using a 32-channel head coil at the Vanderbilt University Institute for Imaging Science. Images were processed on the Vanderbilt University Institute of Imaging Science Center for Computational Imaging XNAT platform (70) in MATLAB (version 2018a; The MathWorks, Inc.) using SPM12 (http://www.fil.ion.ucl.ac.uk/spm). FMRI data was collected with a voxel resolution of 2.5x2.5x2.5, FOV of 96x96, a 1300 millisecond TR, TE of 35 seconds, and a 79 degree flip angle. For the fMRI data, co-registration and distortion correction was conducted using a TOPUP reverse phase encoding scan. Scans were registered to a T1-weighted scan, warped to MNI-space, and spatially smoothed (6mm FWHM). Six motion parameters were included for each run (rotation and translation).

Neuroimaging Analysis
A subset of 35 eligible individuals conducted the 3PRL task during collection of fMRI data. Data processing occurred in SPM12[33] and FSL[34]. Each trial (160 total) was modeled as having a “decision-period” (time between when the cards were seen and a deck was selected) and a “feedback-period” (time between when feedback was received and the next fixation cross was presented). We expected activation during the decision-period to be most relevant for associations with volatility priors, as this is when prior expectations should be influencing deck choice. Scripts for HGF modeling and fMRI pre-processing can be found here: https://github.com/baxpr/prl-fmri https://github.com/baxpr/eprime-3PRL.

Prior to data analysis, masks for the bilateral striatum and PFC were identified as regions of interest (ROIs) using structural masks (2mm Oxford-GSK-Imanova striatal atlas[35]; Neuromorphometrics (http://Neuromorphometrics.com/)) (Figure S3). First, explicit masks were applied to the decision-period across all scans. Clusters within these masks that were significantly activated by the task (FDR p<.05; “thresholded masks”) were then included as explicit masks to test change over time in BOLD activation pre/post treatment, controlling for voxel displacement. Significant clusters showing change over time were identified using 999 cluster-corrected wild-bootstrapping in the Sandwich Estimator Toolbox (SwE) for SPM (http://www.nisox.org/Software/SwE/), which was designed for flexible longitudinal analysis of neuroimaging data and provides robust control of false positive results in unbalanced data[36]. Clusters showing significant change in activation pre/post treatment, at cluster-level p<.005, were examined for relationships with clinical and cognitive variables.

Parameter Recovery
We performed ten simulations per participant using the participant’s actual data (their choices and the corresponding outcome) and their derived set of perceptual parameters.

© 2025 Sheffield JM et al. *JAMA Network Open*

Simulated data based on these subject parameters were then used to recover parameter estimates. Recovered HGF parameters (10 per participant) were then averaged and correlated with the participant's actual parameters from their performance on the task. We observed significant positive correlations between recovered and actual estimates for μ_3^0 (r=.17, p=.04) and ω_3 (r=.31, p=.008) but not κ (r=.13, p=.09) (Figure S2). Unlike in prior reports, our data do demonstrate recoverability of third-level parameters in simulation.

© 2025 Sheffield JM et al. *JAMA Network Open*

eResults

Participants

One participant in the befriending group removed themselves from the study after randomization, but before learning their treatment allocation. Another had a diagnosis of substance-induced psychosis from the SCID. The final intent-to-treat sample included 62 individuals (32 CBTp; 30 befriending) (Table 1). The final MRI sample included 35 individuals (16 CBTp; 19 befriending) who completed at least one MRI (3 were missing pre-treatment data due to scanner error, 6 were missing post-treatment data), met diagnostic criteria and completed at least four sessions of psychotherapy (Table S2). Of the final sample, 75% reported no changes in medication dosage throughout the duration of treatment, with similar rates of medication change across the treatment groups (27% befriending, 23% CBTp; X^2=.66, p=.77).

At the end of treatment, self-reported working alliance did not significantly differ between those in CBTp and befriending, indicating that the treatments elicited similar levels of connection with the therapist and sense that they were working on something useful (F(1,47)=3.2, p=.08, Cohen's d=.52).

Adverse Events

During the study period following randomization, 6 adverse events were recorded across 5 different participants (3 in CBTp; 2 in Befriending). All events involved psychiatric hospitalization of the participant in the context of suicidal ideation and/or worsening psychosis.

Other Belief Updating Metrics

In linear mixed models we observed a significant reduction in sensitivity to volatility (κ) (F=(1,112)=7.1 (p=.009), Cohen's d=.50) but no main effect of treatment (F(1,112)=.46, p=.50) or treatment by time interaction (F(1,112)=.05, p=.82). We also observed an increase in meta-volatility learning rate (ω_3) (F(1,112)=7.0 (p=.009), Cohen's d=.50), but no main effect of treatment (F(1,112)=.01, p=.92) or treatment by time interaction (F(1,112)=.57, p=.45).

No significant association with overall PSYRATS was observed for κ (F(1,107.6)=1.9, p=.17) and ω_3 (F(1,108.6)=.69, p=.41). However, change in κ (F(1,107.3)=6.5, p=.01), and meta-volatility learning rate (F(1,104.4)=15.6, p<.001) were associated with clinical improvement in overall positive symptoms

PANSS Positive Items

In order to determine whether longitudinal associations between PANSS positive symptoms and prior on volatility were related to delusions items on the PANSS, we examined associations between volatility priors and the following items: P1 (delusions), P5 (grandiosity), P6 (suspiciousness), and G9 (unusual thought content). We also looked at relationships with P3 (hallucinations). These items were selected because they have been

© 2025 Sheffield JM et al. *JAMA Network Open*

found to load together in meta-analysis of PANSS items[60]. A sum of the delusion items was calculated and showed a significant association with volatility priors in a linear mixed model (F(1,107.8)=10.1, p=.002, Cohen's d=.61). Volatility priors were not significantly associated with clinical improvement in hallucinations (F(1,98.1)=3.1, p=.08, Cohen's d=.36).

Of the individual delusion items, delusions (F(1,111.2)=5.4, p=.02, Cohen's d=.44) and grandiosity (F(1,112)=9.4, p=.003, Cohen's d=.58) were significantly associated with volatility priors. Significant associations were not observed for suspiciousness (F(1,105)=1.5, p=.22, Cohen's d=.24) or unusual thought content (F(1,101.7)=3.9, p=.05, Cohen's d=.39).

<u>Sensitivity and Specificity Analyses</u>

Healthy Comparison Participants

A group of healthy comparison participants (N=27) were recruited as part of another research study conducted at Vanderbilt University Medical Center. Healthy comparison participants completed the 3PRL task two times in the MRI scanner, 8-weeks apart (information on this cohort and study in the Supplemental Materials). Change in volatility priors and activation in right caudate and left PFC regions were examined in this cohort, to test for normative change over time in the absence of any intervention.

Healthy comparison participants demonstrated no significant change in volatility priors (F(1,50)=.21, p=.65, Cohen's d=.13) and no significant change in activation in the right caudate (F(1,51)=.65, p=.43, Cohen's d=.23) or left PFC (F(1,51)=.34, p=.56, Cohen's d=.16) after 8-weeks (Figure S4).

Depression, negative, and general symptoms

Depressive, negative, and general symptoms were examined to test for specificity of relationships with positive symptoms. Negative symptoms (F(1,113)=5.0, p=.03, Cohen's d=.42), depression (F(1,113)=12.3, p<.001, Cohen's d=.66), and general psychopathology (F(113)=15.8, p<.001, Cohen's d=.75) all improved with psychotherapy. Despite this improvement, volatility priors were not significantly associated with change in depression (F(1,108.8)=1.0, p=.31), negative symptoms (F(1,108.2)=1.2, p=.27), or general symptoms (F(1,110.7)=1.7, p=.68).

Medication

All primary analyses were conducted controlling for antipsychotic dose at the time of the assessment (risperidone equivalence)[41]. All primary analyses continued to demonstrate significant changes over time (volatility priors: F(1,101)=4.6, p=.035; PSYRATS total: F(1,101)=62.1, p<.001; PANSS Positive: F(1,60.3)=27.5, p<.001) and associations between cognitive, brain, and clinical variables with medication included in the model: volatility priors and PANSS positive (F(1,100.1)=8.6, p=.004); volatility priors and caudate activation (F(1,55.6)=13.8, p<.001); lPFC activation and positive symptoms (F(1,35)=15.4, p<.001).

© 2025 Sheffield JM et al. *JAMA Network Open*

Caudate activation and positive symptoms were slightly attenuated with the inclusion of medication (F(1,30.2)=3.1, p=.09); however the correlation between change in caudate activation and positive symptoms remained significant when controlling for medication (r=.42, p=.04).

Whole Brain Analysis
In addition to ROIs, we explored regions across the whole brain that showed a significant reduction in activation with treatment. A list of these regions can be found in Table S3 but include the caudate, cerebellum, hippocampus, intraparietal sulcus, and sensory regions.

For each of these regions, we examined associations with volatility priors. For regions showing a significant association with volatility priors, we then asked whether those regions also relate to PSYRATS and/or PANSS Positive symptoms.

For the two clusters that survived FWE-correction – the right caudate and cerebellum- we observed significant associations with volatility priors (caudate: F(1,59)=8.9, p=.004; cerebellum: F(1,59)=8.1, p=.006) Consistent with our other findings, activation in these regions was also significantly associated with PANSS positive symptoms (caudate: F(1,42.2)=8.0, p=.007; cerebellum: F(1,38.5)=8.3, p=.006), but not PSYRATS total scores (all p's>.09).

In addition to these regions, we also observed significant associations between volatility priors and hippocampal activity (F(1,50.3)=8.5, p=.005). Hippocampal activity was also related to PANSS positive symptoms (F(1,33.1)=6.8, p=.01), but not PSYRATS (F=1,46)=1.9, p=.17).

Reduced Threshold Analysis

Given the uniqueness of this dataset and its relatively small MRI sample size, we explored whether any additional clusters within our ROIs demonstrate a reduction in activation following treatment, at a lower threshold (p<.01, k=15). We found two additional clusters: 1) Reduced activation pre/post treatment in the left caudate [-18 6 20]. Interestingly, this is a very similar location as our right caudate cluster reported in the main text [18 6 22]. Like our findings in the right caudate, activation in this left caudate cluster related to reductions in volatility priors (F(1,59)=4.5, p=.04) and PANSS positive symptoms (F(1,37.1)=5.3, p=.03), but not PSYRATS (p=.13). 2) A cluster in the right dorsolateral prefrontal cortex (rdlPFC) [38 30 40] that showed reduced activation pre/post treatment. Reduced activation in that region was related to reductions in PANSS positive symptoms (F(1,38.3)=4.9, p=.03), but not reduced volatility priors (p=.15) or PSYRATS (p=.79).

Alternative Modeling Approach (Cole Model)
Our main analysis represent an a-priori decision about how to model the behavioral data, that is consistent with our previous work[16–18]. However, since this decision was made, others have published alternative approaches to estimating volatility using the

Hierarchical Gaussian Filter model[61] that has been recently applied to 3PRL data[62]. As such, we estimated our primary outcome of interest, μ_3^0, using the prior mean and variance assumed by this model. We then re-tested our primary outcomes, to determine whether they were consistent across these two models.

We continued to observe a significant reduction in μ_3^0 following treatment (F(1,108)=10.2, p=.002, Cohen's d=.61), with no main effect of treatment (F(1,108)=.11, p=.74) and no significant treatment by time interaction (F(1,108)=.00, p=.98).

Interestingly, under this model, we observed a significant relationship between μ_3^0 and PSYRATS total scores, in support of our primary hypothesis (F(1,103.1)=4.7, p=.03, Cohen's d=.43). μ_3^0 was also still significantly related to PANSS positive symptoms (F(1,84.7)=9.1, p=.003).

Relationships with brain activation were slightly attenuated. Associations between μ_3^0 and caudate activation were no longer significant (F(1,64)=3.4, p=.07, Cohen's d=.46).

© 2025 Sheffield JM et al. *JAMA Network Open*

eTable 1: Outcomes

Outcomes												
	Befriending N=30 pre-treatment N=22 post-treatment		CBTp Intervention N=32 pre-treatment N=28 post-treatment		Main Effect of Time		Main Effect of Treatment		Time*Treatment		Overall Change with Treatment (Main Effect of Time)	Treatment Effect (Treatment by Time Interaction)
**Primary Outcomes**												
**PSYRATS Total**	**Mean (SD)**	**Change**	**Mean (SD)**	**Change**	**F-Statistic**	**p-value**	**F-Statistic**	**p-value**	**F-Statistic**	**p-value**	**Cohen's d [95% CI]**	**Cohen's d [95% CI]**
Baseline	15.5 (4.7)		15.5 (5.0)									
8-weeks	11.6 (7.3)	-3.9	10.8 (6.9)	-4.7	F(1,112)= 59.7	<.001*	F(1,112)= .01	.92	F(1,112)= .06	.81	d= 1.5 [1.0, 1.9]	d= .05 [-.32, .42]
**Prior on Volatility (μ_3^0)**												
Baseline	.13 (1.0)		.21 (1.0)									
8-weeks	-.49 (1.4)	-.62	-.01 (.96)	-.22	F(1,112)= 7.7	.006*	F(1,112)= .23	.63	F(1,112)= .06	.80	d= .52 [.15, .90]	d= .05 [-.32, .42]
**Secondary Outcome – Positive Symptoms**												
**PANSS Positive**												
Baseline	23.6 (5.3)		24.7 (5.5)									
8-weeks	22.4 (6.5)	-1.2	21.8 (6.4)	-2.9	F(1,113)= 14.7	<.001*	F(1,113)= .30	.59	F(1,113)= .17	.68	d= 0.72 [.34, 1.1]	d= .08 [-.29, .45]
**Secondary Outcomes – Sensitivity Analysis, Clinical**												
**PANSS P1 (Delusions)**												
Baseline	5.2 (.85)		5.4 (.95)									
8-weeks	4.9 (1.4)	-0.3	4.8 (1.4)	-0.6	F(1,66.7) =14.1	<.001*	F(1,64.7) =.03	.87	F(1,67.0) =.12	.73	d= 0.92 [.41, 1.4]	d= .08 [-.39, .56]
**PANSS P5 (Grandiosity)**												
Baseline	3.4 (1.8)		2.9 (1.8)									
8-weeks	3.6 (1.6)	0.2	3.0 (2.0)	0.1	F(1,113)= .15	.70	F(1,113)= 0.0	.10	F(1,113)= .24	.63	d=.07 [-.30, .44]	d= .09 [-.28, .46]
**PANSS P6 (Suspiciousness)**												
Baseline	5.5 (.86)		5.6 (.72)									
8-weeks	4.8 (1.2)	-0.7	5.0 (.98)	-0.6	F(1,113)= 32.8	<.001*	F(1,113)= 1.4	.24	F(1,113)= 2.9	.09	d= 1.1 [.68, 1.5]	d= .32 [-.05, .69]

© 2025 Sheffield JM et al. *JAMA Network Open*

**PANSS G9 (Unusual Thought Content)**												
Baseline	2.8 (1.5)		3.2 (1.8)									
8-weeks	3.0 (1.5)	0.2	2.7 (1.7)	-0.5	F(1,55.2) =1.2	.28	F(1,63.2) =.33	.57	F(1,55.3) =1.1	.30	d=.29 [-.24, .82]	d= .28 [-.25, .81]
**PANSS P3 (Hallucinations)**												
Baseline	3.7 (2.1)		4.3 (2.3)									
8-weeks	3.9 (2.3)	0.2	3.5 (2.1)	-0.8	F(1,113)= 10.4	.002*	F(1,113)= .38	.54	F(1,113)= 2.2	.14	d= .61 [.23, .98]	d= .28 [-.09, .65]
**BDI-II**												
Baseline	21.4 (13.5)		25.2(15.8)									
8-weeks	19.8 (14.2)	-1.6	16.2 (14.8)	-9.3	F(1,110)= 12.3	<.001*	F(1,110)= 1.3	.25	F(1,110)= 5.0	.03	d= .67 [.28, 1.1]	d= .43 [.05, .80]
**PANSS Negative**												
Baseline	14.2 (4.9)		14.9 (5.1)									
8-weeks	13.1 (4.7)	-1.1	14.3 (4.4)	-0.6	F(1,113)= 5.0	.03	F(1,113)= .52	.47	F(1,113)= .11	.74	d= .49 [.04, -1.1]	d= .06 [-.31, .43]
**PANSS General**												
Baseline	37.8 (5.3)		40.5 (8.0)									
8-weeks	37.6 (7.5)	-0.2	34.0 (8.3)	-5.5	F(1,113)= 15.8	<.001*	F(1,113)= 2.7	.10	F(1,113)= 4.8	.03	d=.75 [.36, 1.1]	d= .41 [.04, .78]
**Secondary Outcomes – Sensitivity Analysis, Computational Parameters**												
**Sensitivity to Volatility (κ)**												
Baseline	.54 (.09)		.55 (.07)									
8-weeks	.50 (.08)	-.04	.52 (.08)	-.03	F(1,112)= 7.1	.009*	F(1,112)= .04	.84	F(1,112)= .05	.82	d= .50 [.13, .88]	d= .04 [-.33, .41]
**Meta-volatility Learning Rate (ω_3)**												
Baseline	-1.2 (.90)		-1.4 (.81)									
8-weeks	-1.1 (.78)	-.1	-1.1 (.73)	-.3	F(1,112)= 7.0	.009*	F(1,112)= .01	.92	F(1,112)= .57	.45	d= .50 [.12, .88]	d= .14 [-.23, .51]

© 2025 Sheffield JM et al. *JAMA Network Open*

*significant, including after Bonferroni-correction for multiple comparisons. Primary outcomes are not corrected, however for secondary outcomes the critical p-value was $p<.006$ (clinical outcomes) or $p<.025$ (computational outcomes)

© 2025 Sheffield JM et al. *JAMA Network Open*

eTable 2: MRI Cohort

Demographic	CBTp Intervention N=16	Befriending N=19	Comparison
Age	34.8 (14.6)	30.3 (10.2)	F=1.1, p=.29
Sex (M/F/O)	10/5/1	13/6/0	X^2=1.2, p=.54
Race (W/B/O)	9/6/1	10/8/1	X^2=2.1, p=.55
Personal Education	14.1 (2.1)	13.5 (1.8)	F=.68, p=.41
Parental Education	14.4 (3.1)	15.1 (3.0)	F=.44, p=.51
Premorbid IQ	101.2 (13.2)	98.5 (15.7)	F=.27, p=.61
Cognitive Ability	-1.2 (.77)	-1.4 (1.3)	F=.48, p=.49
Risperidone Equiv	4.2 (2.3)	5.6 (3.5)	F=1.31, p=.26
PSYRATS Total	17.4 (3.3)	16.9 (3.2)	F=.22, p=.64
PANSS Positive	24.1 (5.4)	23.7 (5.4)	F=.03, p=.86

© 2025 Sheffield JM et al. *JAMA Network Open*

eTable 3: Significant clusters in whole brain analysis

Pre>Post: Reduced Activation Over Time<br>Cluster-wise p<.001, voxel-wise p<.001, k>15				
	**MNI Coordinates**	**cluster-size**	**Peak z-value**	**p-value**
**Decision-Period**				
Caudate	16 32 4	42	4.54	pFWEcorr=.04
Cerebellum	-34 -68 -42	60	3.60	pFWEcorr=.03
Caudate	16 34 4<br>16 24 -2	66	4.3	.001
Cerebellum	-20 -66 -32	348	3.89	.001
	32 -38 30	27	3.67	.001
Hippocampus	-32 -30 -2	15	3.31	.001
Intraparietal Sulcus	26 -60 56	44	2.84	.001
Visual Cortex	-20 -90 2	29	2.73	.001
Sensorimotor Cortex	34 -40 66	16	2.71	.001
Visual Cortex	-28 -86 16	15	2.64	.001
Visual Cortex	26 -80 8	15	2.60	.001
**Feedback-Period**				
	None			

Post>Pre: Increased Activation Over Time				
	**MNI Coordinates**	**cluster-size**	**Peak t-value**	**p-value**
**Decision-Period**				
	None			
**Feedback-Period**				
	None			

© 2025 Sheffield JM et al. *JAMA Network Open*

eFigure 1: A) task, B) Decision and feedback-periods defined for fMRI analysis, C) HGF model

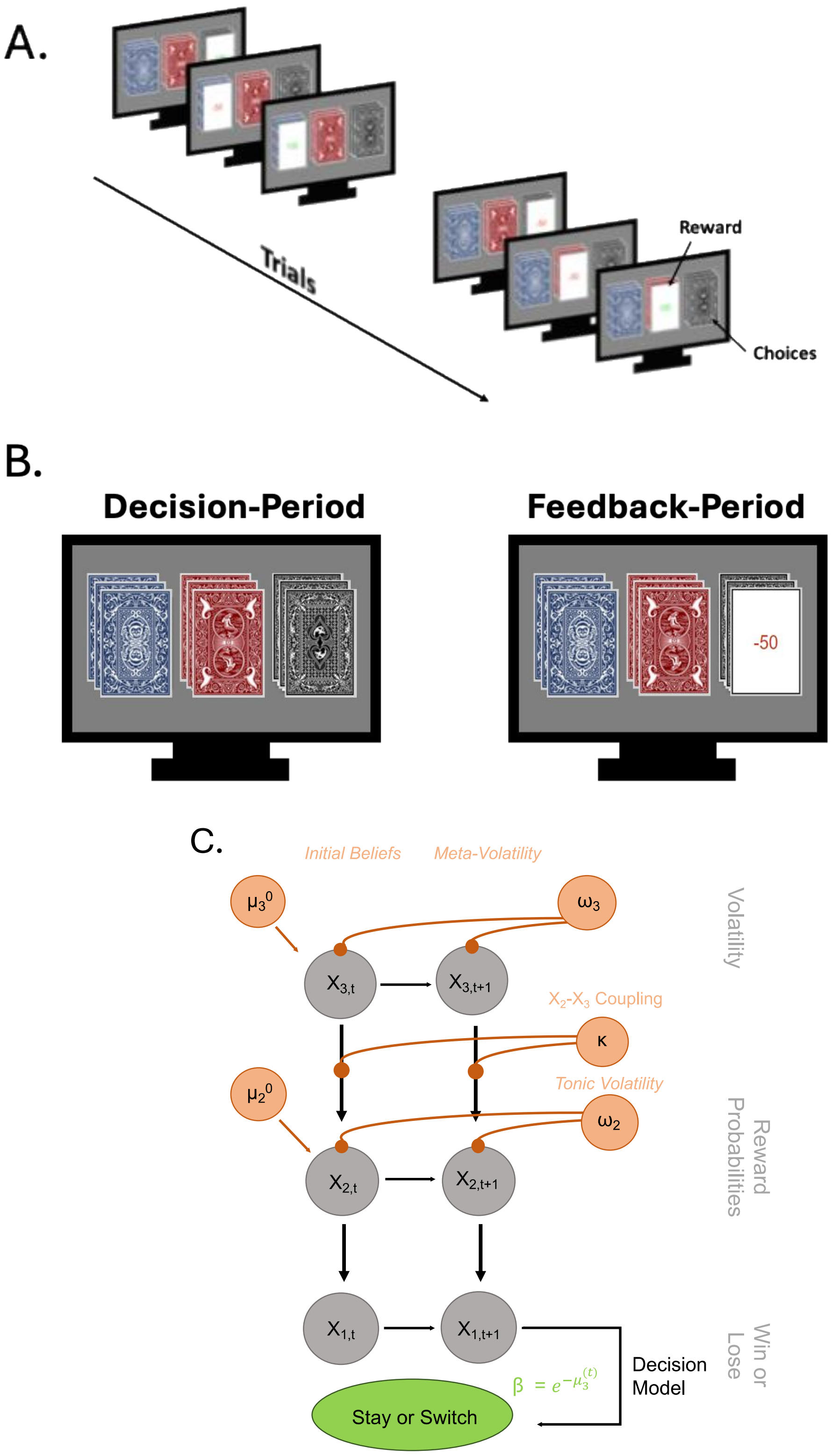

© 2025 Sheffield JM et al. *JAMA Network Open*

eFigure 2: Parameter Recovery

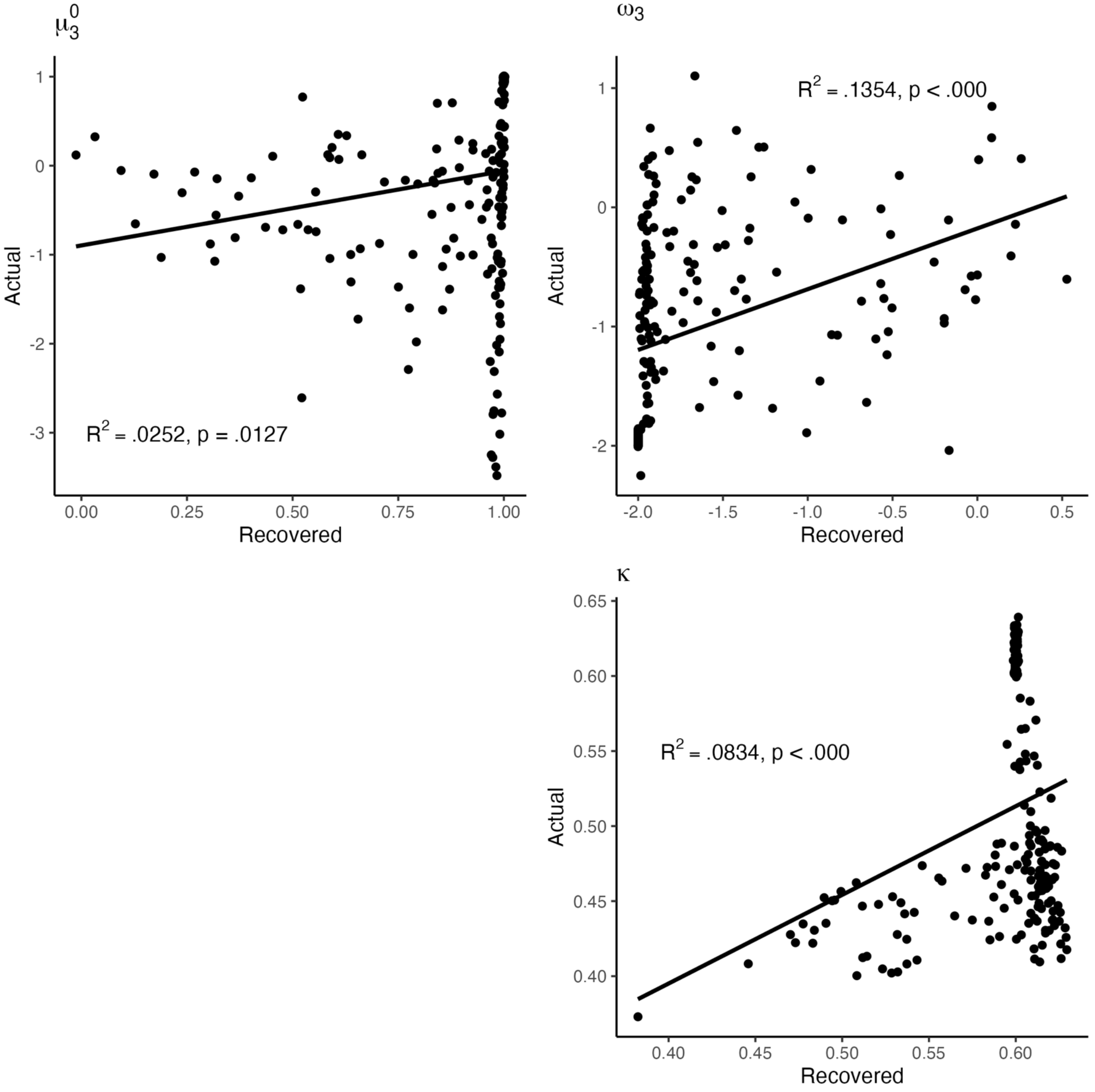

Ten simulations per participant were performed using the participant's actual data (their choices and the corresponding outcome) and their derived set of perceptual parameters. Simulated data based on these subject parameters were then used to recover parameter estimates. Recovered HGF parameters (10 per participant) were then averaged and correlated with the participant's actual parameters from their performance on the task. We observed significant positive correlations between recovered and actual estimates for μ_3^0 (r=.16, p=.01) and ω_3 (r=.36, p<.001) but not κ (r=.28, p<.001).

eFigure 3: Masks used

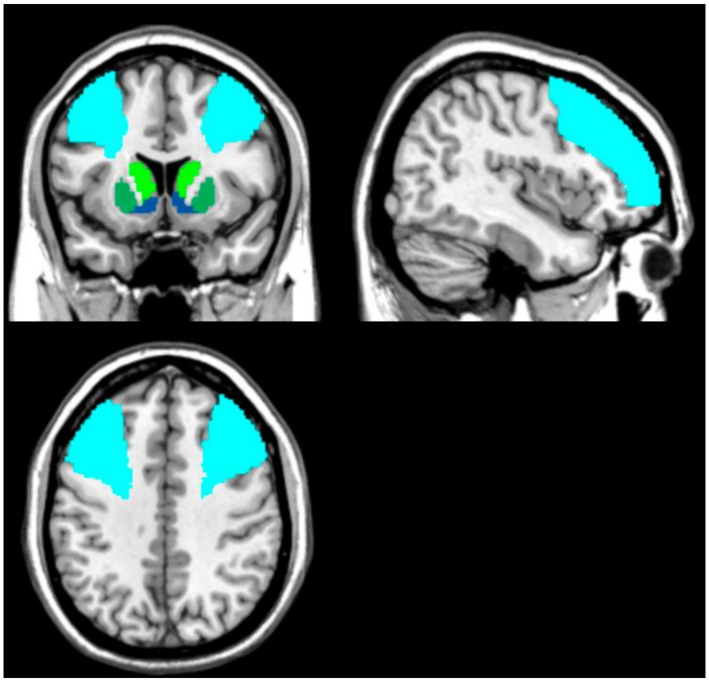

© 2025 Sheffield JM et al. *JAMA Network Open*

eFigure 4: Healthy Comparison Group

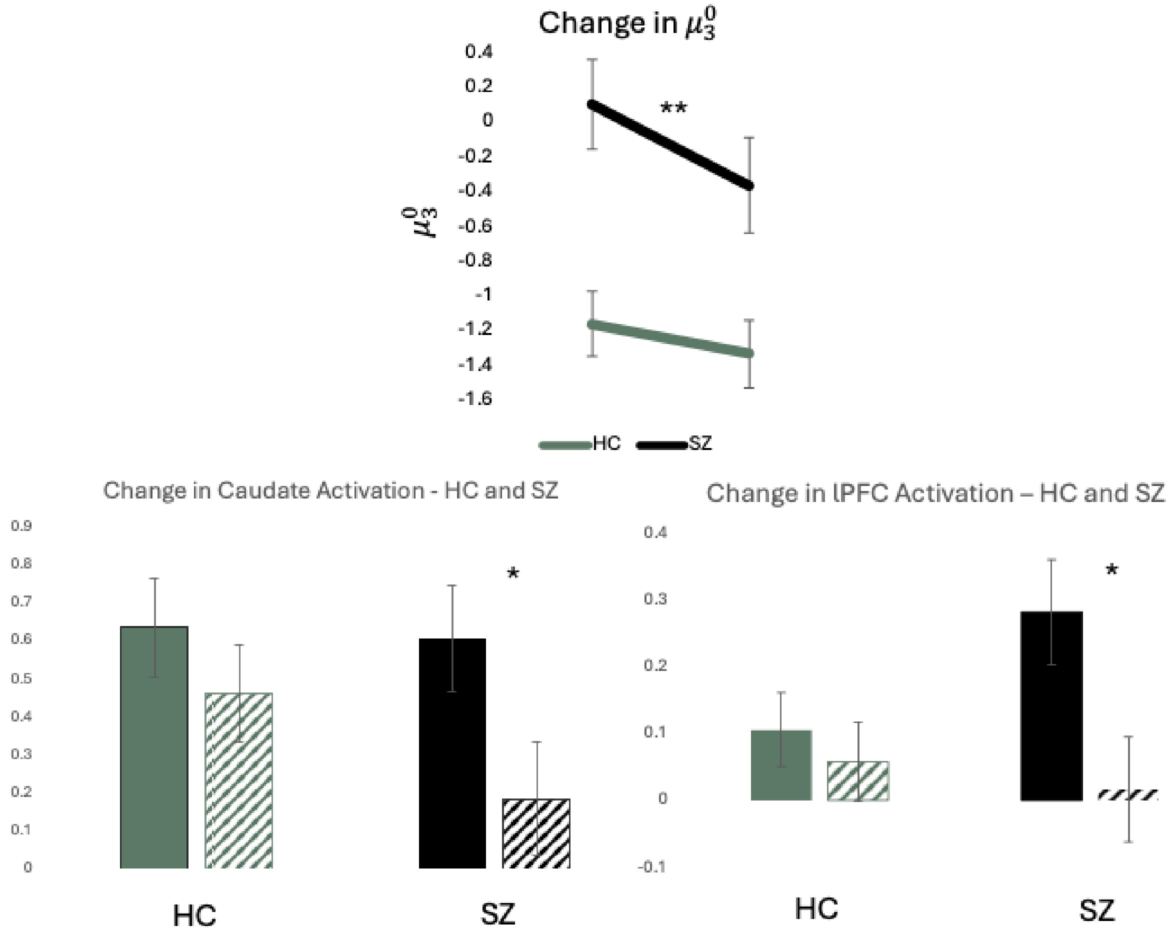

Healthy comparison participants who completed the 3PRL task twice in the fMRI scanner, 8-weeks apart, demonstrated no significant change in volatility priors, caudate activation and lPFC activation. In contrast, individuals with schizophrenia (N=35) undergoing psychotherapy revealed significant reductions in volatility priors, caudate and lPFC activation during decision-making.

© 2025 Sheffield JM et al. *JAMA Network Open*
