# Supplementary material for: Prior Expectations of Volatility Following Psychotherapy for Delusions: A Randomized Clinical Trial
Source: JAMA Netw Open. 2025 Jun 24;8(6):e2517132. doi: 10.1001/jamanetworkopen.2025.17132 (PMC12188364; doi:10.1001/jamanetworkopen.2025.17132)
Supplement: Supplement 3. — Data Sharing Statement [file jamanetwopen-e2517132-s003.pdf]

Data Sharing Statement

Sheffield. Prior Expectations of Volatility Following Psychotherapy. *JAMA Netw Open*.
Published June 24, 2025. doi:10.1001/jamanetworkopen.2025.17132

Data
Additional Information: NCT04748679
Data available: No
